# Lkb1 maintains T_reg_ cell lineage identity

**DOI:** 10.1038/ncomms15876

**Published:** 2017-06-16

**Authors:** Di Wu, Yuechen Luo, Wei Guo, Qing Niu, Ting Xue, Fei Yang, Xiaolei Sun, Song Chen, Yuanyuan Liu, Jingru Liu, Zhina Sun, Chunxiao Zhao, Huifang Huang, Fang Liao, Zhongchao Han, Dongming Zhou, Yongguang Yang, Guogang Xu, Tao Cheng, Xiaoming Feng

**Affiliations:** 1 State Key Laboratory of Experimental Hematology, Institute of Hematology and Hospital of Blood Diseases, Chinese Academy of Medical Sciences & Peking Union Medical College, Tianjin 300020, China; 2 Department of Medical Microbiology, Tongji Medical College, Huazhong University of Science and Technology, Wuhan 430030, China; 3 Central Laboratory, The Union Hospital of Fujian Medical University, 29 Xinquan Road, Fuzhou 350001, China; 4 Institute Pasteur of Shanghai, Shanghai Institutes for Biological Sciences, Chinese Academy of Sciences, Shanghai 200031, China; 5 First Hospital of Jilin University, Changchun 130012, China; 6 Nanlou Respiratory Department, Chinese PLA General Hospital, Beijing 100853, China

## Abstract

Regulatory T (T_reg_) cells are a distinct T-cell lineage characterized by sustained *Foxp3* expression and potent suppressor function, but the upstream dominant factors that preserve T_reg_ lineage-specific features are mostly unknown. Here, we show that Lkb1 maintains T_reg_ cell lineage identity by stabilizing *Foxp3* expression and enforcing suppressor function. Upon T-cell receptor (TCR) stimulation Lkb1 protein expression is upregulated in T_reg_ cells but not in conventional T cells. Mice with T_reg_ cell-specific deletion of Lkb1 develop a fatal early-onset autoimmune disease, with no Foxp3 expression in most T_reg_ cells. Lkb1 stabilizes *Foxp3* expression by preventing STAT4-mediated methylation of the conserved noncoding sequence 2 (CNS2) in the *Foxp3* locus. Independent of maintaining *Foxp3* expression, Lkb1 programs the expression of a wide spectrum of immunosuppressive genes, through mechanisms involving the augmentation of TGF-β signalling. These findings identify a critical function of Lkb1 in maintaining T_reg_ cell lineage identity.

## Abstract (web summary)

The protein kinase Lkb1 has been shown to limit conventional T cell activation and pro-inflammatory functions. Here the authors show that Lkb1 also maintains Foxp3 expression and suppressive function in regulatory T (T_reg_) cells, and that T_reg_-specific Lkb1-deficient mice develop fatal autoimmune disease.

Regulatory T (T_reg_) cells preserve immune homeostasis by suppressing autoreactive immune responses^1^,^2^. The T_reg_ cell lineage can be defined by two basic characteristics, stable expression of the transcription factor Foxp3 (forkhead box P3) and potent suppressive capacity^3^,^4^. T_reg_ cells are stable and usually retain lineage characteristics *in vivo*^5^,^6^. However, under some conditions, T_reg_ cells can lose or alter their lineage identity, resulting in immune disturbance and development of diseases^3^,^4^,^6^,^7^. Thus, delineating the molecular mechanisms that maintain the T_reg_ lineage identity is important for understanding and treating T_reg_ cell-related immune diseases.

Foxp3 is the most specific marker for distinguishing T_reg_ cells from other T cells and an important regulator required for programming T_reg_ suppressive function^1^,^2^. Lineage tracing in mice has shown that thymus-derived mature T_reg_ cells have relatively stable Foxp3 expression in both homeostasis and inflammatory conditions^5^. Other reports indicate that epigenetic demethylation of the conserved noncoding sequence 2 (CNS2, also known as T_reg_ cell-specific demethylation region) in the *Foxp3* locus can ensure stable Foxp3 expression in T_reg_ cells^8^,^9^,^10^. However, the upstream signalling checkpoints that activate chromatin, and the demethylation status, of *Foxp3* locus are not clear.

T_reg_ cells suppress immune responses through diverse mechanisms, such as the modulation of antigen presentation function (via CTLA4 (cytotoxic T-lymphocyte associated protein 4)), the production of inhibitory cytokines (for example, interleukin (IL)-10 and IL-35) and metabolites (for example, reactive oxygen species and adenosine), the deprivation of the T-cell growth factor IL-2 (via CD25), and the direct killing of target cells (via granzyme B and perforin)^2^,^11^,^12^. Although Foxp3 has an important function in programming T_reg_ cells by controlling the expression of a large number of immunosuppressive genes, Foxp3 alone is not sufficient to confer this function^1^,^2^. In addition, T-cell receptor (TCR) signalling is also important to promote T_reg_ cell function^13^,^14^,^15^. Nevertheless, little is known about other upstream ‘master regulators’ that broadly control the expression of these T_reg_ cell-associated immunosuppressive genes.

Liver kinase b1 (Lkb1) is a tumour suppressor, and is mutated in Peutz–Jeghers cancer syndrome, cervical carcinoma and many sporadic non-small-lung carcinomas^16^,^17^,^18^. Under energy-stressed conditions, Lkb1 is an important upstream kinase that phosphorylates AMP-activated protein kinase (AMPK) and AMPK-related kinases that coordinate cell growth with metabolism^16^,^17^,^18^. Lkb1 has been shown to restrain the activation and proinflammatory function of conventional T cells^18^,^19^.

In this study, we find that Lkb1 protein is specifically increased in T_reg_ cells upon TCR stimulation. To understand the function and mechanism of Lkb1 in T_reg_ cells, we generate a mouse line with Lkb1 specifically deleted in T_reg_ cells. These mice develop a fatal early-onset autoimmune disease with defective maintenance of stable *Foxp3* expression and suppressive capacity in T_reg_ cells. Mechanistically, Lkb1 restrains STAT4 (signal transducer and activator of transcription 4) activation partially through suppressing nuclear factor-κB (NF-κB) signalling, and thus prevents STAT4-mediated methylation of CNS2 in the *Foxp3* locus, resulting in stable Foxp3 expression. Meanwhile, Lkb1 promotes the expression of a large number of immunosuppressive genes partially through augmenting transforming growth factor-β (TGF-β) signalling. Our study identifies Lkb1 as a critical determinant of T_reg_ cell linage identity.

## Results

### Lkb1 protein is increased in T_reg_ cells upon TCR stimulation

**Figure 1:**
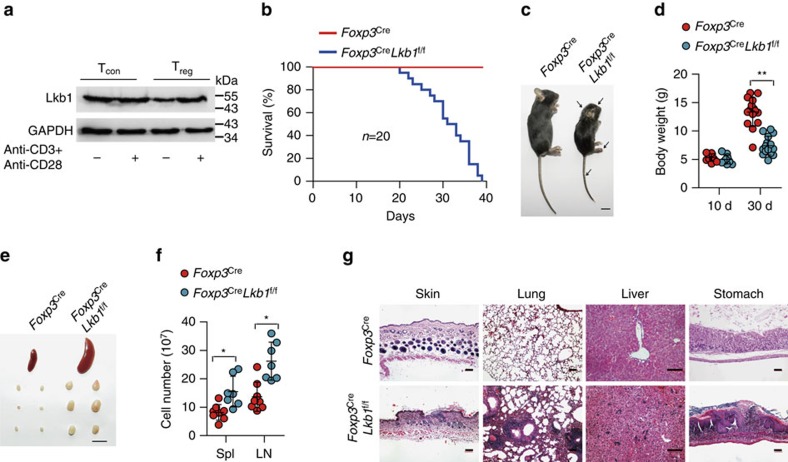
T_reg_ cell-specific deletion of Lkb1 leads to a fatal autoimmune disease. (**a**) Lkb1 proteins in CD4^+^YFP^−^ conventional T cells and CD4^+^YFP^+^ T_reg_ cells untreated or stimulated in plates coated with anti-CD3 and anti-CD28 in the presence of IL-2 for 24 h determined by western blot. (**b**) Survival of *Foxp3*^Cre^ and *Foxp3*^Cre^*Lkb1*^f/f^ mice (*n*=20). (**c**) A representative appearance of 30-day-old *Foxp3*^Cre^ and *Foxp3*^Cre^*Lkb1*^f/f^ mice (scale bar, 1 cm). (**d**) Body weight of *Foxp3*^Cre^ and *Foxp3*^Cre^*Lkb1*^f/f^ male mice of different ages (*n*=10–15). (**e**) A representative picture of spleen and lymph nodes from 30-day-old *Foxp3*^Cre^ and *Foxp3*^Cre^*Lkb1*^f/f^ mice. (scale bar, 1 cm). (**f**) Total cell numbers in spleen (Spl) and lymph nodes (LNs) of *Foxp3*^Cre^ and *Foxp3*^Cre^*Lkb1*^f/f^ mice (*n*=7–8). (**g**) Representative haematoxylin and eosin-stained skin, lung, liver and stomach sections from *Foxp3*^Cre^ and *Foxp3*^Cre^*Lkb1*^f/f^ mice (scale bar, 100 μm). All mice analysed were 28–30 days old, unless otherwise specified. Log-rank survival curve was used for survival analysis in **b**, and two-way analysis of variance (ANOVA) was used for statistical analyses in **d**,**f** (*P<0.05, **P<0.01, ****P<0.0001); error bars represent s.d.; all data are representative of at least three independent experiments.

TCR stimulation is essential for T_reg_ cells to exert their optimal function^13^,^14^,^15^. Lkb1 protein expression in T_reg_ cells were slightly lower compared with conventional T cells without stimulation (Fig. 1a and Supplementary Figs 1a and 8a,b). However, upon TCR stimulation, Lkb1 protein expression was markedly upregulated in T_reg_ but not conventional T cells (Fig. 1a and Supplementary Fig. 1a), implying that Lkb1 might be particularly important for T_reg_ cells to execute their immunoregulatory effect.

### Deletion of Lkb1 in T_reg_ cells leads to a fatal autoimmunity

**Figure 2:**
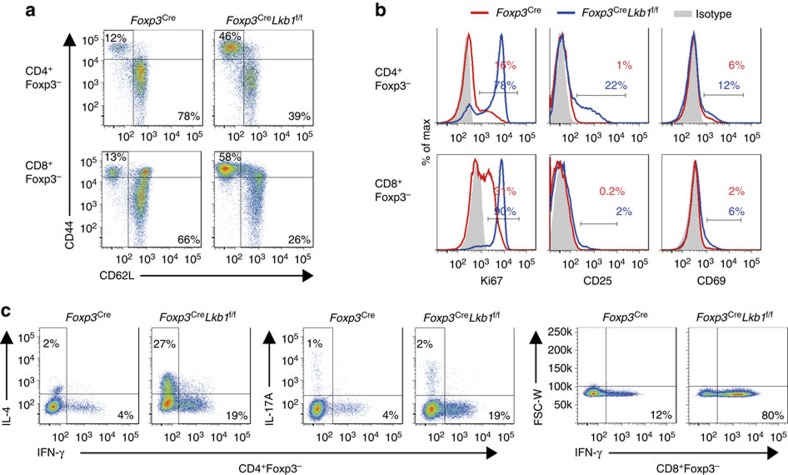
Spontaneous activation of conventional T cells in *Foxp3*^Cre^*Lkb1*^f/f^ mice. (**a**) Expression of CD44 and CD62L on splenic CD4^+^Foxp3^−^ and CD8^+^Foxp3^−^ T cells from *Foxp3*^Cre^ and *Foxp3*^Cre^*Lkb1*^f/f^ mice. (**b**) Expression of Ki67, CD25 and CD69 in splenic CD4^+^Foxp3^−^ and CD8^+^Foxp3^−^ T cells from *Foxp3*^Cre^ and *Foxp3*^Cre^*Lkb1*^f/f^ mice. (**c**) Intracellular staining of cytokines in splenic CD4^+^Foxp3^−^ and CD8^+^Foxp3^−^ T cells from *Foxp3*^Cre^ and *Foxp3*^Cre^*Lkb1*^f/f^ mice stimulated with phorbol myristate acetate (PMA) and ionomycin for 4 h. All mice analysed were 28–30 days old. All data are representative of at least three independent experiments.

To investigate the role of Lkb1 in T_reg_ cells, we generated a mouse line with Lkb1 conditionally depleted in T_reg_ cells by crossing *Foxp3*^YFP-Cre^ (*Foxp3*^Cre^, which ensure T_reg_ cell-specific deletion of a target gene) mice^20^ with *Lkb1*^f/f^ mice^21^. Lkb1 protein was depleted in CD4^+^YFP^+^ T_reg_ cells from *Foxp3*^Cre^*Lkb1*^f/f^ mice (Supplementary Figs 1b and 8c,d). Strikingly, loss of Lkb1 in T_reg_ cells caused early moribund at ∼35 days of age (Fig. 1b). *Foxp3*^Cre^*Lkb1*^f/f^ mice exhibited smaller size, decreased mobility, hunched posture, collapsed ears and tail skin lesions (Fig. 1c), and barely increased in body weight with age (Fig. 1d). *Foxp3*^Cre^*Lkb1*^f/f^ mice displayed splenomegaly and lymphadenopathy (Fig. 1e), and they had increased cell numbers in secondary lymphoid organs (Fig. 1f). Histopathological analysis revealed massive infiltration of lymphocytes into multiple organs such as skin, lung, liver and stomach (Fig. 1g), suggesting T-cell autoimmunity. In support of this, *Foxp3*^Cre^*Lkb1*^f/f^ mice had substantial expansion of CD4^+^ conventional T cells (Supplementary Fig. 1c), associated with drastically increased percentages of cells that displayed a CD44^high^CD62L^low^ effector/memory phenotype (Fig. 2a and Supplementary Fig. 1d). These mice also had higher percentages of CD4^+^ and CD8^+^ conventional T cells that expressed the proliferation marker Ki67 and acute activation markers CD25 and CD69 (Fig. 2b and Supplementary Fig. 1e), and produced the inflammatory cytokines interferon-γ, IL-4 and IL-17A (Fig. 2c and Supplementary Fig. 1f). These phenotypes were similar in severity to those observed in mice deficient of Foxp3 (ref. 22) or depleted of T_reg_ cells^23^, indicating a severe defect of immune suppression mediated by T_reg_ cells.

### Lkb1 maintains *Foxp3* expression and CNS2 demethylation

**Figure 3:**
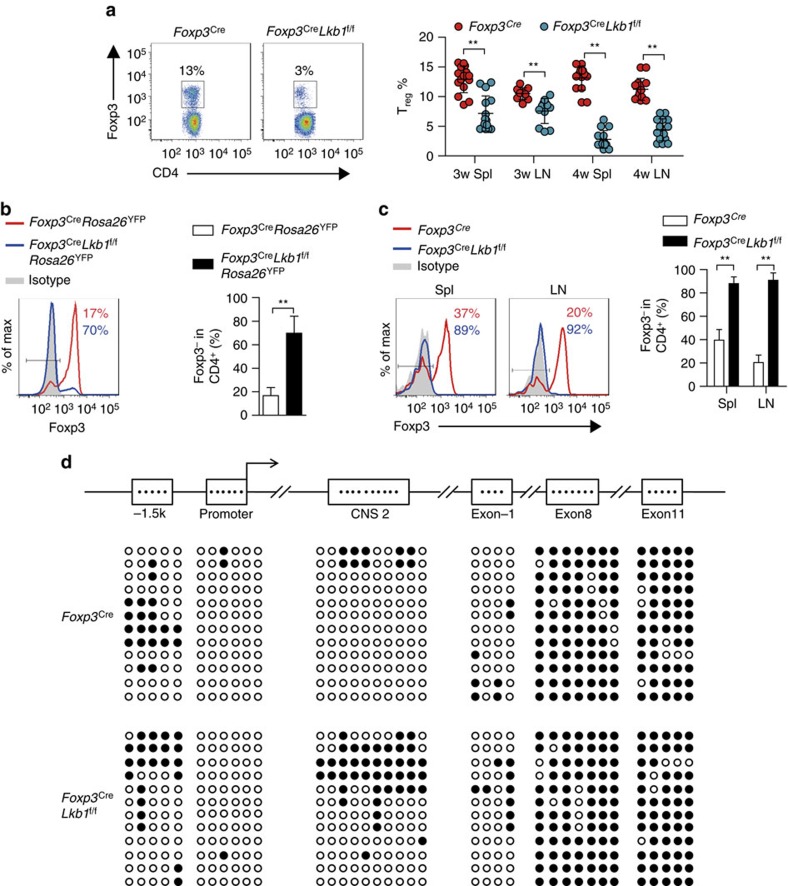
Lkb1 stabilizes *Foxp3* expression and prevents CNS2 methylation. (**a**) Foxp3^+^ cell percentages among CD4^+^ T cells in spleen and lymph nodes from *Foxp3*^Cre^ and *Foxp3*^Cre^*Lkb1*^f/f^ mice of different ages (*n*=10–15). (**b**) Foxp3 expression in CD4^+^*Rosa26*-YFP^high^ (*Rosa26*-YFP labelled the cells that have experienced the expression of Foxp3, *Rosa26*-YFP was much brighter than and thus distinguishable from Foxp3-YFP) cells from *Foxp3*^Cre^ *Rosa26*^YFP^ and *Foxp3*^Cre^*Lkb1*^f/f^ *Rosa26*^YFP^ mice. (**c**) Foxp3 expression in T_reg_ cells doubly sorted from *Foxp3*^Cre^ and *Foxp3*^Cre^*Lkb1*^f/f^ mice and transferred together with T_n_ cells into *Rag1*^−/−^ mice for 3 weeks (*n*=3). (**d**) Methylation of the CpG motifs of the −1.5 kb region, promoter, CNS2, exon 1, exon 8 and exon 11 across the *Foxp3* locus in T_reg_ cells from *Foxp3*^Cre^ and *Foxp3*^Cre^*Lkb1*^f/f^ mice, determined by bisulfite sequencing. Filled circles represent methylated CpG sites and open circles represent unmethylated CpG sites. Two-way analysis of variance (ANOVA) was used for statistical analyses in **a,c**, and unpaired two-tailed Student’s *t*-test was used for statistical analyses in **b** (**P<0.01); error bars represent s.d.; all data are representative of at least two independent experiments.

T_reg_ cells normally stay at a relatively constant abundance within the CD4^+^ T-cell population, and expand concomitantly with effector/memory T cells to preserve immune homeostasis during inflammation^24^. Although not altered at early ages, the percentages of peripheral T_reg_ cells among CD4^+^ T-cell populations continuously dropped to ∼3% by 4 weeks of age when the mice became moribund (Fig. 3a). Despite greatly increased numbers of effector/memory T cells in *Foxp3*^Cre^*Lkb1*^f/f^ mice (Supplementary Fig. 1d), the absolute numbers of T_reg_ cells were only comparable with those in wild-type mice (Supplementary Fig. 1g), suggesting that Lkb1-deficient T_reg_ cells fail to accumulate concomitantly with effector/memory T cells in *Foxp3*^Cre^*Lkb1*^f/f^ mice at later ages that might contribute to the autoimmunity in *Foxp3*^Cre^*Lkb1*^f/f^ mice.

The survival and proliferation of Lkb1-deficient T_reg_ cells were barely altered compared with wild-type control cells (Supplementary Fig. 2a,b). Thus, other reasons might account for the defective accumulation of Lkb1-deficient T_reg_ cells. T_reg_ cells are considered a stable lineage marked by sustained Foxp3 expression^5^, but under certain conditions they might lose Foxp3 expression and develop into cells resembling effector T cells (designated ex-T_reg_ cells)^25^. To investigate whether Lkb1-deficient T_reg_ cells were prone to lose Foxp3 expression, we introduced the *Rosa26*^YFP^ allele (a loxp-site-flanked STOP cassette followed by the YFP-encoding sequence was inserted into the *Rosa26* locus, and the expression of YFP from the *Rosa26* locus was dependent on the expression of Cre recombinase) into wild-type and conditional knockout mice^26^. CD4^+^*Rosa26*-YFP^high^ cells (100% Lkb1-deficient T_reg_ cells) sorted from *Foxp3*^Cre^*Lkb1*^f/f^*Rosa26*^YFP^ mice contain much higher percentages of Foxp3^−^ cells (which represented ex-T_reg_ cells that ever experienced Foxp3 expression but ceased to express it later) than that from *Foxp3*^Cre^*Rosa26*^YFP^ mice (Fig. 3b). Therefore, a large proportion of Lkb1-deficient T_reg_ cells lost Foxp3 expression and became ex-T_reg_ cells that might result in the decrease of T_reg_ cell abundance *in vivo*.

Foxp3 is an X-chromosome-encoded transcription factor. Due to random X inactivation, female mice heterozygous for *Foxp3*^*Cre*^ (*Foxp3*^Cre/+^) should contain ∼1:1 ratio of *Foxp3*^*Cre*^-positive T_reg_ cells to *Foxp3*^*Cre*^-negative T_reg_ cells. Hence, the *Foxp3*^Cre^-heterozygous female *Foxp3*^Cre/+^*Lkb1*^f/f^ mice were devoid of autoimmune diseases due to the presence of T_reg_ cells lacking *Foxp3*^Cre^ expression but retaining a wild-type Lkb1 allele^8^. Proinflammatory cytokines may impair T_reg_ cell stability^3^,^6^. To determine whether the instability of T_reg_ cells was due to either the inflammatory environment in *Foxp3*^Cre^*Lkb1*^f/f^*Rosa26*^YFP^ mice or the intrinsic defect of the cells, we utilized *Foxp3*^Cre^-heterozygous female *Foxp3*^Cre/+^*Lkb1*^f/f^*Rosa26*^YFP^ mice that were devoid of autoimmune diseases. Despite the presence of wild-type T_reg_ cells devoid of the expression of *Foxp3*^Cre^ in *Foxp3*^Cre/+^*Lkb1*^f/f^*Rosa26*^YFP^ mice, the CD4^+^*Rosa26*-YFP^high^ cells from these mice were cells that had ever experienced the expression of *Foxp3*^Cre^ that resulted in the deletion of Lkb1 and expression of Rosa26-YFP. Similarly, we observed substantially higher proportions of Foxp3^−^ cells among sorted CD4^+^*Rosa26*-YFP^high^ cells from *Foxp3*^Cre/+^*Lkb1*^f/f^*Rosa26*^YFP^ mice than that from *Foxp3*^Cre/+^*Rosa26*^YFP^ mice (Supplementary Fig. 2c), ruling out the possibility that overproduction of proinflammatory cytokines in *Foxp3*^Cre^*Lkb1*^f/f^ mice were the causative reasons for T_reg_ cell instability.

Foxp3^−^*Rosa26*-YFP^+^ ex-T_reg_ cells might also arise from recently activated T cells that transiently expressed Foxp3 but did not develop into a full T_reg_ program (which were also considered as poorly committed T_reg_ cells)^27^. To determine whether the full committed T_reg_ cells lose stability, we doubly sorted CD4^+^CD25^+^YFP^+^ cells from CD45.1^+^CD45.2^+^ wild-type *Foxp3*^Cre^ and CD45.1^−^CD45.2^+^*Foxp3*^Cre^*Lkb1*^f/f^ mice, mixed them together with congenitally marked CD45.1^+^ CD45.2^−^ naive T (T_n_) cells and transferred them into *Rag1*^−/−^ mice. Strikingly, we observed that nearly all the Lkb1-deficient T_reg_ cells lost Foxp3 expression 3 weeks after transfer (Fig. 3c), despite the fact that most wild-type T_reg_ cells retained Foxp3 expression. This was not due to the contamination of non-T_reg_ cells because the starting population contained >99.5% Foxp3^+^ T_reg_ cells after double sorting (Supplementary Fig. 2d). These results confirm that Foxp3 expression is lost in a large proportion of fully committed mature Lkb1-deficient T_reg_ cells, rather than in a subset of poorly committed Foxp3^+^ cells.

Next, we investigated the molecular mechanisms by which Lkb1 promoted T_reg_ cell stability. Because Foxp3 protein expression level was barely altered in Lkb1-deficient T_reg_ cells (Supplementary Fig. 2e), Lkb1 seemed to not control T_reg_ stability through affecting Foxp3 protein production or degradation. The epigenetic status across the *Foxp3* locus has been shown to be critical for sustained Foxp3 expression^9^. Two recent reports demonstrated that deletion of CNS2 region in the *Foxp3* locus led to the loss of T_reg_ cell stability^8^,^10^, suggesting that CNS2 is pivotal for maintaining stable Foxp3 expression in T_reg_ cells. CNS2 is completely demethylated in T_reg_ cells, but not in conventional T cells^9^,^28^,^29^,^30^. T_reg_ cell lineage development is partially dependent on the demethylation of CNS2 (refs 31, 32), and CNS2 has enhancer activity that is markedly reduced after methylation^31^. Therefore, we examined the methylation of CpG motifs across the *Foxp3* locus by bisulfite sequencing in CD4^+^YFP^+^ T_reg_ cells from *Foxp3*^Cre^*Lkb1*^f/f^ mice and *Foxp3*^Cre^ mice, and found a significant increase (nearly 25%) in methylation of CpG at CNS2 in Lkb1-deficient T_reg_ cells *ex vivo* (Fig. 3d). T_reg_ cells that already lost Foxp3 expression were excluded in this analysis. Although *Foxp3* promoter and other potentially regulatory regions are associated with T_reg_ cell lineage identity^29^,^33^,^34^, the methylation of CpG at sites including −1.5 kb region, promoter, exon 1, exon 8 and exon 11 across the *Foxp3* locus in Lkb1-deficient T_reg_ cells was comparable to the wild-type T_reg_ cells (Fig. 3d). Therefore, we hypothesize that methylation of CpG at CNS2 may contribute to the instability of Lkb1-deficient T_reg_ cells. It seems that the methylation of CNS2 is not a secondary effect of Foxp3 reduction, because Foxp3 protein level was barely altered in Lkb1-deficient T_reg_ cells that already had substantial CNS2 methylation. Given the critical role of CNS2 in conferring T_reg_ cell stability established by previous studies, our results together suggested that CNS2 methylation might lead to the instability of Lkb1-deficient T_reg_ cells.

### Lkb1 functions in T_reg_ cells independent of AMPK

AMPK is the well-known Lkb1 downstream target critical for coordinating metabolism^13^. Interestingly, we found no obvious decrease of AMPK activity in Lkb1-deficient Treg cells (Supplementary Figs 3a and 8e–h). Therefore, other kinases such as calmodulin-dependent protein kinase kinase-β (CamKKβ) and transforming growth factor-β-activated kinase-1 (TAK1)^15^ may promote AMPK activation in T_reg_ cells. These data argue that Lkb1 is not important for AMPK activation in T_reg_ cells, and Lkb1 substrates other than AMPK are responsible for the observed phenotype of Lkb1-deficient T_reg_ cells. Consistent with this, T_reg_ cells that lacked the expression of AMPKα1/2 (from *Foxp3*^Cre^*AMPKα1*^f/f^*AMPKα2*^f/f^ mice, Supplementary Fig. 3b) did not exhibit any adverse phenotype and *in vivo* functional impairment (Supplementary Fig. 3c,d).

### Lkb1 prevents STAT4 activation and STAT4 binding to CNS2

**Figure 4:**
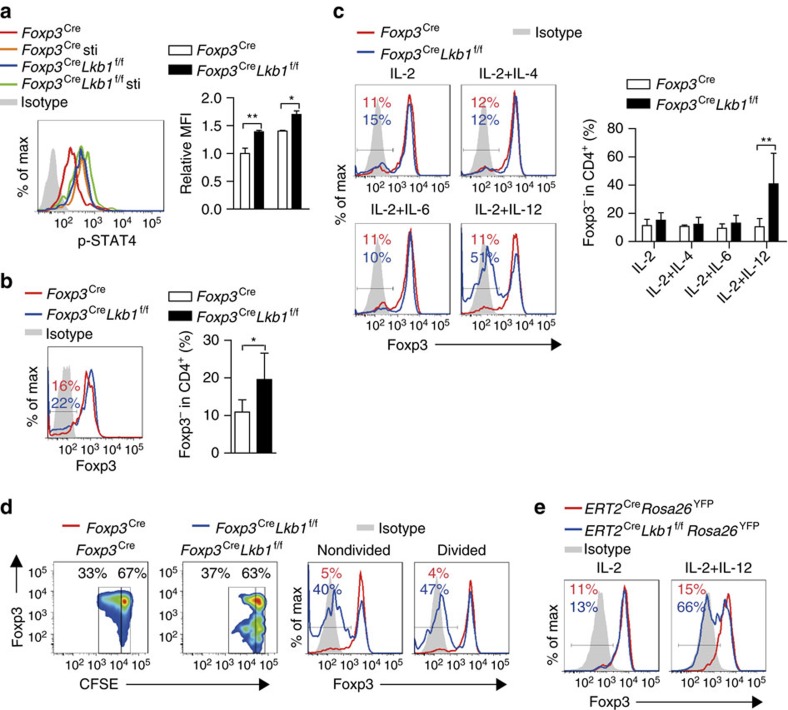
Lkb1 prevents STAT4 activation to maintain T_reg_ cell stability. (**a**) Intracellular phosphorylated STAT4 in *Foxp3*^Cre^ and *Foxp3*^Cre^*Lkb1*^f/f^ T_reg_ cells treated with or without IL-12 (*n*=3). (**b**) Foxp3 expression in *Foxp3*^Cre^ and *Foxp3*^Cre^*Lkb1*^f/f^ T_reg_ cells co-cultured with DCs without any cytokines (*n*=7). (**c**) Foxp3 expression in *Foxp3*^Cre^ and *Foxp3*^Cre^*Lkb1*^f/f^ T_reg_ cells co-cultured with DCs supplemented with indicated cytokines (*n*=3). (**d**) Foxp3 expression in divided and nondivided *Foxp3*^Cre^ and *Foxp3*^Cre^*Lkb1*^f/f^ T_reg_ cells co-cultured with DCs for 4 days supplemented with IL-2 and IL-12. (**e**) Foxp3 expression in *ERT2*^Cre^*Rosa26*^YFP^ and *ERT2*^Cre^*Lkb1*^f/f^*Rosa26*^YFP^ T_reg_ cells co-cultured with DCs supplemented with indicated cytokines and 4-hydroxytamoxifen. Two-way analysis of variance (ANOVA) was used for statistical analyses in **a**,**c**, and unpaired two-tailed Student’s *t*-test was used for statistical analyses in **b** (*P<0.05, **P<0.01); error bars represent s.d.; all data are representative of at least two independent experiments.

STAT3 and STAT6 are respectively the downstream mediators of the inflammatory cytokines IL-6 and IL-4 that bind to *Foxp3* gene to mediate CpG methylation and T_reg_ cell instability^8^, whereas the T_reg_ cell growth factor IL-2 signals via STAT5 to counteract the destabilizing effect of these inflammatory cytokines^8^. Therefore, we examined whether the STAT family protein activations were altered in Lkb1-deficient T_reg_ cells. Although STAT3 and STAT5 phosphorylations were barely affected, we detected a drastic increase of STAT4 phosphorylation and a slight increase of STAT6 phosphorylation in Lkb1-deficient T_reg_ cells with or without the stimulation of relevant cytokines (Fig. 4a and Supplementary Figs 4a,b and 8i–k). The *in vivo* maintenance of T_reg_ cells depends on their contact with dendritic cells (DCs)^35^,^36^, and hence we developed an *in vitro* DC-T_reg_ cell co-culture system that could mimic the physiological situation to test which signalling would cause the instability of T_reg_ cells. Lkb1-deficient T_reg_ cells slightly lost stability when co-cultured with DCs without exogenous cytokines (Fig. 4b). Treatment with IL-2 alone, or IL-2 in combination with IL-4 or IL-6, did not lead to a significant loss of Foxp3 protein expression in Lkb1-deficient T_reg_ cells after 4 days of culture (Fig. 4c). Strikingly, addition of IL-12, a potent STAT4 activator, caused the loss of Foxp3 expression in more than 50% Lkb1-deficient but not wild-type T_reg_ cells (Fig. 4c), suggesting that STAT4 hyperactivation is the causative reason for the instability of Lkb1-deficient T_reg_ cells. In addition, the apoptosis and proliferation were barely changed in Lkb1-deficient T_reg_ cells compared with wild-type T_reg_ cells (Supplementary Fig. 4c,d), suggesting that the loss of Foxp3 expressing Lkb1-deficient T_reg_ cells in response to IL-2 + IL-12 is not due to their altered apoptosis or proliferation. Intriguingly, both divided and nondivided Lkb1-deficient T_reg_ cells lost their stability (Fig. 4d). To determine whether the instability of Lkb1-deficient T_reg_ cells was due to an intrinsic signalling alteration or *in vivo* selection of the T_reg_ subpopulations with reduced stability, we used *ERT2*^Cre^*Lkb1*^f/f^*Rosa26*^YFP^ mice in which Lkb1 could be induced to be acutely deleted in T_reg_ cells (Supplementary Figs 4e and 8l,m), thus avoiding the long-term *in vivo* selection. A similar IL-12-induced loss of stability was observed in T_reg_ cells after induced Lkb1 deletion with 4-hydroxytamoxifen *in vitro* (Fig. 4e), implying an intrinsic role for STAT4 hyperactivation in destabilizing Lkb1-deficient T_reg_ cells.

**Figure 5:**
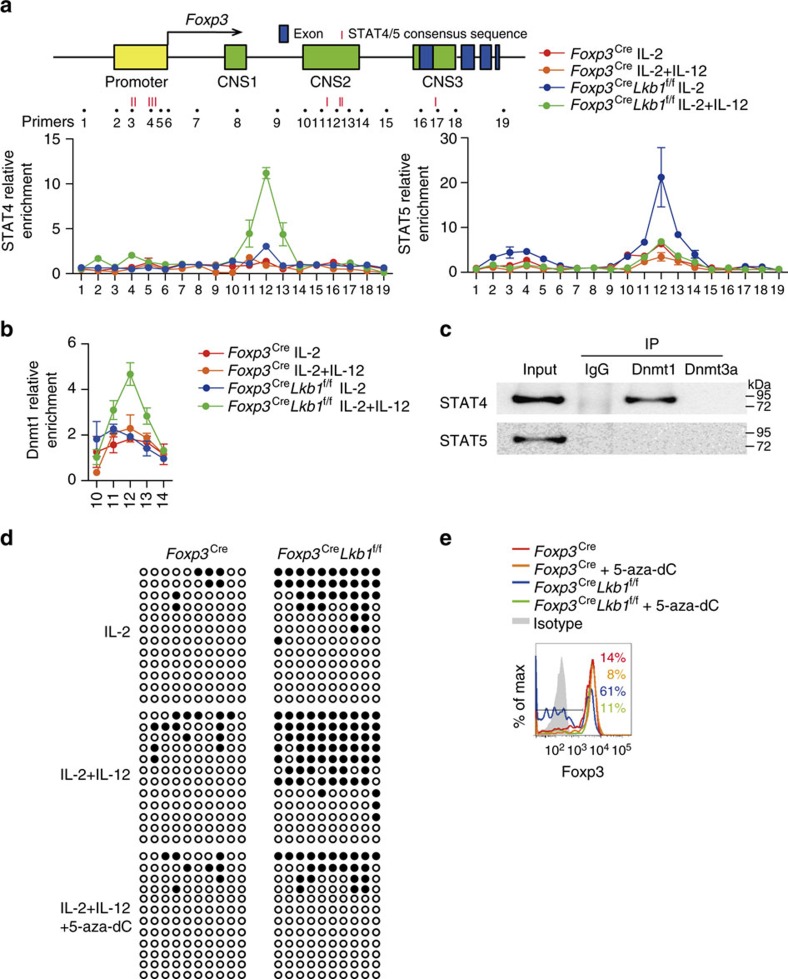
Lkb1 prevents STAT4 binding to CNS2. (**a**) Binding of STAT4 and STAT5 to the *Foxp3* locus in *Foxp3*^Cre^ and *Foxp3*^Cre^*Lkb1*^f/f^ T_reg_ cells treated with IL-2 or IL-2 plus IL-12, determined by ChIP. (**b**) Binding of Dnmt1 to the CNS2 of *Foxp3* locus in *Foxp3*^Cre^ and *Foxp3*^Cre^*Lkb1*^f/f^ T_reg_ cells treated with IL-2 or IL-2 plus IL-12, determined by ChIP. (**c**) STAT4 and STAT5 coprecipitation with Dnmt1 and Dnmt3a was analysed by using nuclear extract from *in vitro* expanded T_reg_ cells. (**d**) Methylation of the CpG motifs of the *Foxp3* promoter and CNS2 in *Foxp3*^Cre^ and *Foxp3*^Cre^*Lkb1*^f/f^ T_reg_ cells determined by bisulfite sequencing, and T_reg_ cells were treated with indicated reagents. Filled circles represent methylated CpG sites and open circles represent unmethylated CpG sites. (**e**) Foxp3 expression in *Foxp3*^Cre^ and *Foxp3*^Cre^*Lkb1*^f/f^ T_reg_ cells co-cultured with DCs supplemented with IL-2 plus IL-12 in the presence or absence of 5-aza-dC. All data are representative of at least two independent experiments.

We then tested whether STAT4 could bind to and induce methylation of the *Foxp3* locus in Lkb1-deficient T_reg_ cells. Chromatin immunoprecipitation (ChIP) experiments showed that Lkb1-deficient T_reg_ cells had significantly more STAT4 binding to the CNS2 than did the wild-type cells in response to IL-2 and IL-12 (Fig. 5a and Supplementary Datas 1 and 2). Additionally, compared with IL-2 treatment alone, IL-2 and IL-12 treatment decreased STAT5 binding to the CNS2 in Lkb1-deficient T_reg_ cells (Fig. 5a), suggesting that STAT4 might outcompete STAT5 in DNA binding in the *Foxp3* locus. Since DNA methyltransferase (Dnmt) 1 and 3a were critical for maintaining and inducing DNA methylation respectively^37^, we tested whether STAT4 was capable of recruiting Dnmt1 or 3a to the *Foxp3* locus. Lkb1-deficient T_reg_ cells had significantly more Dnmt1 binding to the CNS2 than did the wild-type cells in response to IL-2 and IL-12 (Fig. 5b), consistent with the results of STAT4 (Fig. 5a). Notably, STAT4 associated with Dnmt1 but not Dnmt3a in T_reg_ cells (Fig. 5c and Supplementary Fig. 8n,o). Furthermore, we examined the methylation of CNS2 in *Foxp3*^Cre^ and *Foxp3*^Cre^*Lkb1*^f/f^ T_reg_ cells treated with IL-2, IL-2+IL-12 or IL-2+IL-12+5-aza-deoxycytidine (5-aza-dC, a DNA methyltransferase inhibitor) to investigate the relationship between STAT4 and CNS2 methylation. In *Foxp3*^Cre^*Lkb1*^f/f^ T_reg_ cells, the methylation of CNS2 was higher after the treatment of IL-2+IL-12 compared with the treatment of IL-2 that could be reversed by 5-aza-dC (Fig. 5d). Indeed, the addition of 5-aza-dC rescued the loss of Foxp3 expression in Lkb1-deficient T_reg_ cells (Fig. 5e). Since 5-aza-dC changes the global DNA methylation status, leading to the global changes in gene expression, we cannot exclude its effect on the expression of other genes. Nevertheless, these results together suggest that Lkb1 maintains stable Foxp3 expression in T_reg_ cells under inflammatory conditions by preventing STAT4-mediated recruitment of Dnmt1 to and subsequent methylation of the *Foxp3* CNS2.

### Lkb1 restrains STAT4 and NF-κB signalling

**Figure 6:**
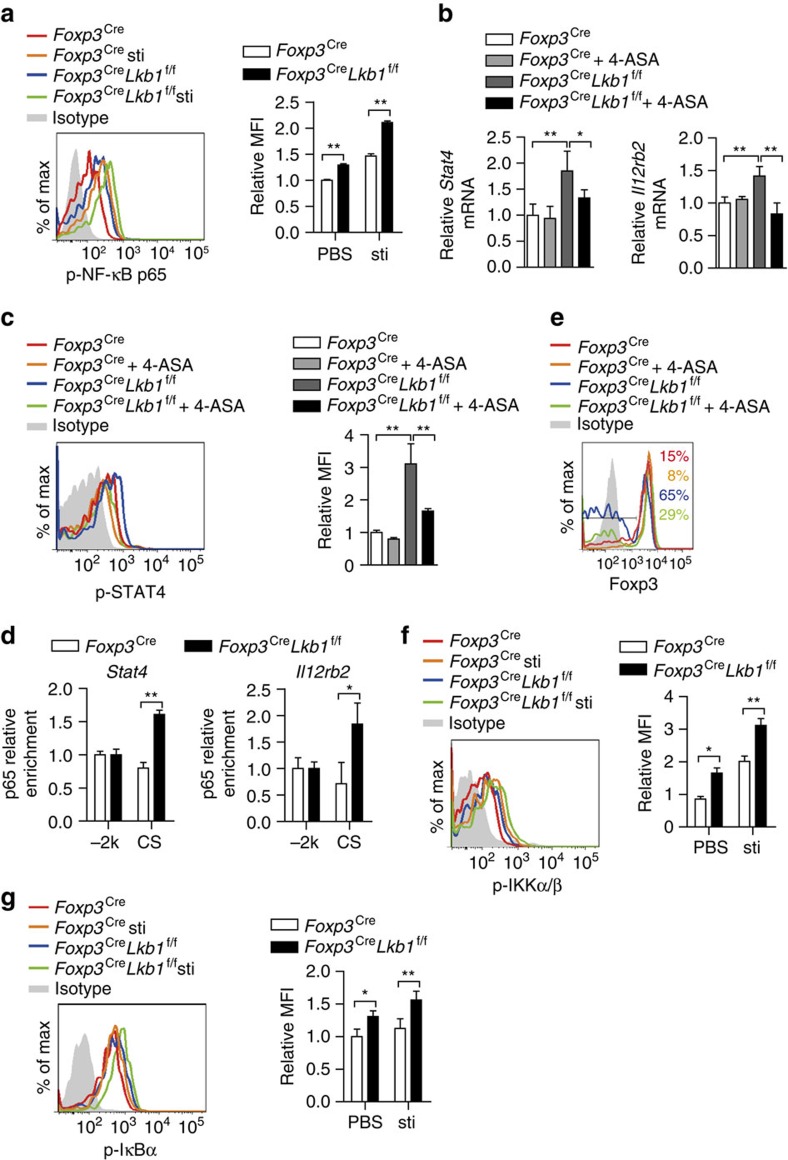
Lkb1 suppresses NF-κB signalling to restrain STAT4 activation. (**a**) Intracellular phosphorylated NF-κB p65 in *Foxp3*^Cre^ and *Foxp3*^Cre^*Lkb1*^f/f^ T_reg_ cells with or without IL-2 stimulation (*n*=3). (**b**) *Stat4* and *Il12rb2* mRNA in *Foxp3*^Cre^ and *Foxp3*^Cre^*Lkb1*^f/f^ T_reg_ cells supplemented with or without 4-ASA for 16 h (*n*=3). (**c**) Intracellular phosphorylated STAT4 in *Foxp3*^Cre^ and *Foxp3*^Cre^*Lkb1*^f/f^ T_reg_ cells supplemented with or without 4-ASA (*n*=3). (**d**) P65 enrichment to consensus sequences (CS) in *Stat4* and *Il12rb2* locus in *Foxp3*^Cre^ and *Foxp3*^Cre^*Lkb1*^f/f^ T_reg_ cells determined by ChIP. (**e**) Foxp3 expression in *Foxp3*^Cre^ and *Foxp3*^Cre^*Lkb1*^f/f^ T_reg_ cells co-cultured with DCs supplemented with IL-2 and IL-12, with or without 4-ASA. (**f**) Intracellular phosphorylated IKKα (Ser176/180)/β (Ser177/181) in *Foxp3*^Cre^ and *Foxp3*^Cre^*Lkb1*^f/f^ T_reg_ cells with or without IL-2 stimulation (*n*=3). (**g**) Intracellular phosphorylated IκBα (Ser 32/36) in *Foxp3*^Cre^ and *Foxp3*^Cre^*Lkb1*^f/f^ T_reg_ cells with or without IL-2 stimulation (*n*=3). Two-way analysis of variance (ANOVA) was used for statistical analyses in (**a**,**b**,**c**,**d**,**f**,**g**) (*P<0.05, **P<0.01); error bars represent s.d.; all data are representative of at least two independent experiments.

The *Stat4* and *Il12rb2* mRNA and IL-12Rβ2 protein expression levels were elevated in Lkb1-deficient T_reg_ cells that could explain the increased STAT4 phosphorylation in these cells in response to IL-12 (Supplementary Fig. 5a,b). To determine which signalling promotes *Stat4* and *Il12rb2* expression in Lkb1-deficient T_reg_ cells, we examined the activation of NF-κB and AKT, the major signalling mediators driving T helper type cell development^38^,^39^. We found that NF-κB p65 but not AKT was hyperactivated in Lkb1-deficient T_reg_ cells (Fig. 6a and Supplementary Figs 5c,d and 8p–r), and the chemical inhibitors to NF-κB reduced *Stat4* and *Il12rb2* mRNA expression (Fig. 6b), as well as STAT4 phosphorylation (Fig. 6c). In addition, conserved NF-κB binding sites were present at the *Stat4* and *Il12rb2* loci (Supplementary Data 1), and ChIP experiments demonstrated more binding of NF-κB p65 to *Stat4* and *Il12rb2* in Lkb1-deficient T_reg_ cells than in wild-type cells (Fig. 6d and Supplementary Datas 1 and 2), suggesting a direct role of NF-κB activation in promoting *Stat4* and *Il12rb2* transcription. Of note, chemical inhibitors of NF-κB could partially rescue the instability of Lkb1-deficient T_reg_ cells (Fig. 6e). These results collectively indicate an involvement of NF-κB activation in driving STAT4 activation and instability of Lkb1-deficient T_reg_ cells.

IκB (inhibitor of NF-κB) kinase (IKK) phosphorylates IκB, leading to their degradation and activation of transcription factor NF-κB. IKK is composed of three subunits, the similar protein kinases IKKα and IKKβ, and a regulatory subunit IKKγ^40^. The phosphorylations serines (Ser) in IKKα (Ser 176/180)/β (Ser 177/181) and IκBα (Ser 32/36) were all increased in Lkb1-deficient T_reg_ cells (Fig. 6f,g), suggesting that Lkb1 might dampen NF-κB signalling by inhibiting IKKα/β activation.

### Lkb1 promotes the expression of immunosuppressive genes

**Figure 7:**
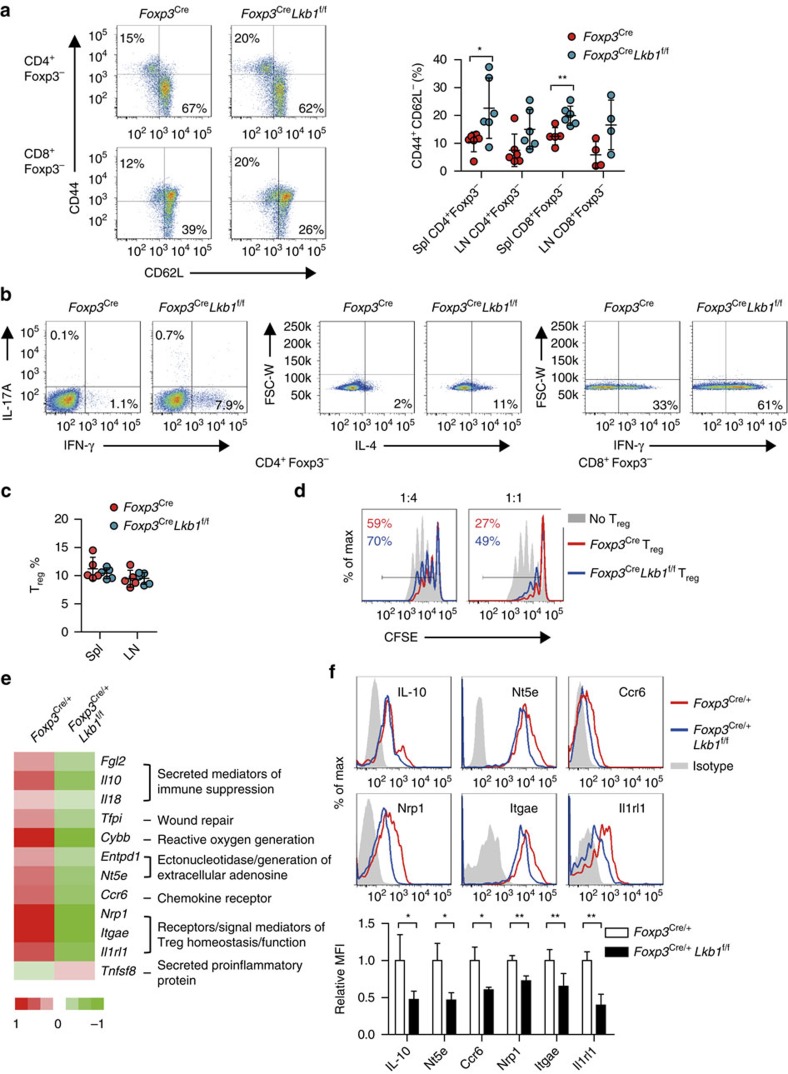
Lkb1 promotes the expression of diverse immunosuppressive genes. (**a**) CD44^high^CD62L^low^ effector/memory cells among splenic CD4^+^Foxp3^−^ and CD8^+^Foxp3^−^ T cells from 9–11-day-old *Foxp3*^Cre^ and *Foxp3*^Cre^*Lkb1*^f/f^ mice (*n*=4–6). (**b**) Intracellular staining of cytokines in splenic CD4^+^Foxp3^−^ and CD8^+^Foxp3^−^ T cells from 9–11-day-old *Foxp3*^Cre^ and *Foxp3*^Cre^*Lkb1*^f/f^ mice stimulated with phorbol myristate acetate (PMA) and ionomycin for 4 h. (**c**) Percentages of Foxp3^+^ T_reg_ cells among CD4^+^ T cells in the spleen and lymph nodes from 9–11-day-old *Foxp3*^Cre^ and *Foxp3*^Cre^*Lkb1*^f/f^ mice (*n*=5). (**d**) Suppression of proliferation of CFSE-labelled T_n_ cells (responding cells, T_resp_) by different ratios of CD4^+^YFP^+^ T_reg_ cells from *Foxp3*^Cre^ and *Foxp3*^Cre^*Lkb1*^f/f^ mice. T_resp_cell division was determined by CFSE dilution at the indicated ratios of cell numbers between T_reg_ cell and T_resp_ cells. The experiment was repeated three times. (**e**) T_reg_ cell function-related genes differentially expressed between CD4^+^YFP^+^ T_reg_ cells from *Foxp3*^Cre/+^ and *Foxp3*^Cre/+^*Lkb1*^f/f^ mice determined by transcriptional profiling, shown in groups based on their functions. Fold difference means fold change of gene expression levels in T_reg_ cells from *Foxp3*^Cre^*Lkb1*^f/f^ mice compared with that from *Foxp3*^Cre^ mice. (**f**) Expression of indicated proteins on splenic T_reg_ cells from *Foxp3*^Cre/+^ and *Foxp3*^Cre/+^*Lkb1*^f/f^ mice (*n*=3). Two-way analysis of variance (ANOVA) was used for statistical analyses in (**a**, **c**, **f**) (*P<0.05, **P<0.01); error bars represent s.d.; all data are representative of at least two independent experiments.

We further explored whether there are defects in Lkb1-deficient T_reg_ cells that did not lose Foxp3 expression yet. The T-cell activation phenotype was already obvious (Fig. 7a,b) in 9–11-day-old *Foxp3*^Cre^*Lkb1*^f/f^ mice that had normal percentages of T_reg_ cells among CD4^+^ T cells (Fig. 7c), suggesting that the suppressor function of Lkb1-deficient T_reg_ cells might be impaired. Indeed, *in vitro* T_reg_ suppression assay showed that with different T_reg_ to T_n_ ratios, Lkb1-deficient T_reg_ cells could not efficiently suppress the proliferation of responder T cells (Fig. 7d), indicating an impaired suppressive capacity of Lkb1-deficient T_reg_ cells.

To investigate the molecular mechanisms by which Lkb1 controls T_reg_ cell function, we compared the transcriptional profiling of CD4^+^YFP^+^ T_reg_ cells sorted from *Foxp3*^Cre/+^ mice with that from *Foxp3*^Cre/+^*Lkb1*^f/f^ mice lacking autoimmune diseases, thus avoiding the secondary impact of the inflammatory environment on T_reg_ cell gene expression (Supplementary Data 3). Kyoto Encyclopedia of Genes and Genomes (KEGG) pathway analysis revealed that the most altered pathways were enriched for genes regulating immune function (Supplementary Fig. 6a). Intriguingly, Lkb1-deficient T_reg_ cells have reduced expression of a wide variety of genes critically involved in T_reg_ cell suppressor function including those encoding secreted molecules associated with immune suppression (*Fgl2*, *Il10* and *Il18*)^11^,^41^, factors in wound-healing processes (*Tfpi*)^11^, regulators of reactive oxygen generation (*Cybb*)^11^, enzymes catalysing the generation of adenosine that directly inhibit proliferation of effector T cells (*Entpd1* and *Nt5e*)^11^,^42^, chemokine receptors critical for T_reg_ cell migration (*Ccr6*)^43^ and cell surface molecules facilitating T_reg_ cell suppressor functions in either tumour environment (*Nrp1*)^44^ or organ-specific inflammatory conditions (*Itgae* and *Il1rl1*)^45^,^46^ (Fig. 7e and Supplementary Fig. 6b). On the other hand, the loss of Lkb1 promoted the expression of genes encoding proinflammatory mediators (*Tnfsf8*)^11^ (Fig. 7e and Supplementary Fig. 6b). It was further confirmed that Lkb1-deficient T_reg_ cells from *Foxp3*^Cre/+^*Lkb1*^f/f^ mice had decreased protein levels of IL-10, Nt5e, Ccr6, Nrp1, Itgae and Il1rl1 (Fig. 7f). Most gene expression alterations were also recapitulated in T_reg_ cells from *Foxp3*^Cre^*Lkb1*^f/f^ mice (Supplementary Fig. 6b). These results clearly showed that Lkb1 promoted the immunosuppressive programs in T_reg_ cells.

### TGF-β signalling in Lkb1-implemented T_reg_ cell function

**Figure 8:**
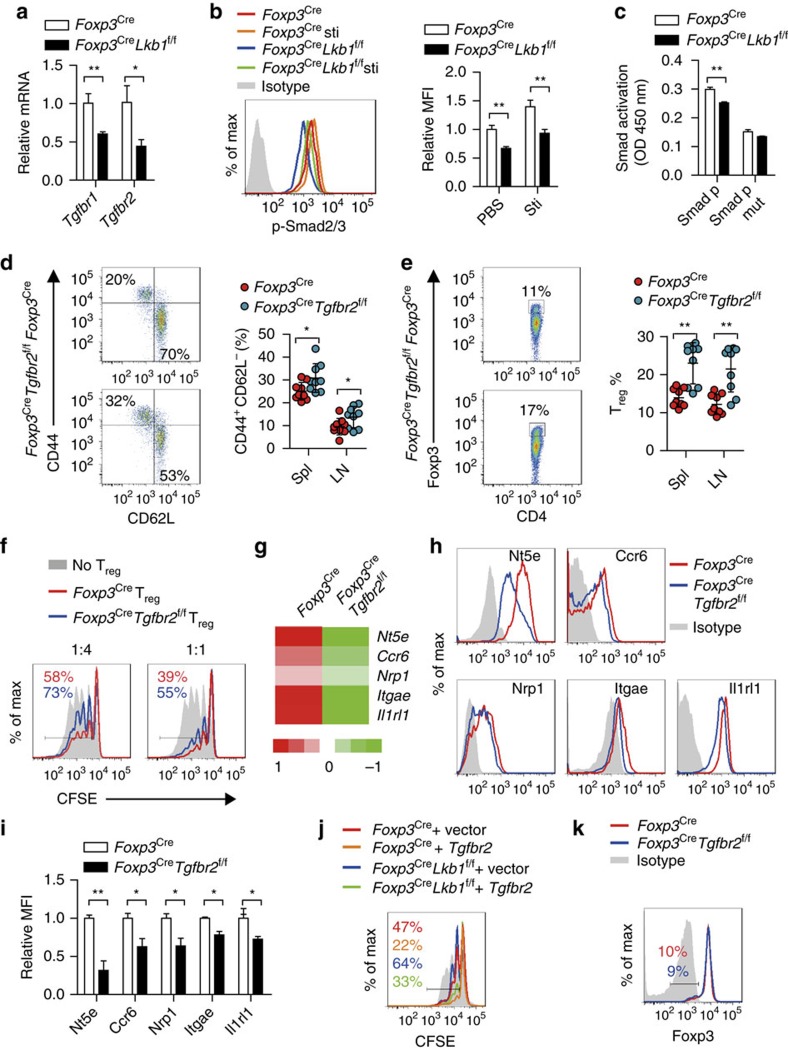
TGF-β signalling is involved in Lkb1-implemented T_reg_ cell suppressor function. (**a**) *Tgfbr1* and *Tgfbr2* mRNA expression in *Foxp3*^Cre^ and *Foxp3*^Cre^*Lkb1*^f/f^ T_reg_ cells by real-time PCR (*n*=3). (**b**) Intracellular expression of phosphorylated Smad2/3 in T_reg_ cells from *Foxp3*^Cre^ and *Foxp3*^Cre^*Lkb1*^f/f^ mice, with or without TGF-β stimulation (*n*=3). (**c**) Binding capacity of Smad2/3 to recognized DNA sequences in nuclear lysates extracted from T_reg_ cells from *Foxp3*^Cre^ and *Foxp3*^Cre^*Lkb1*^f/f^ mice. (**d**) CD44 and CD62L expression on CD4^+^Foxp3^−^ T cells from 6-week-old *Foxp3*^Cre^ and *Foxp3*^Cre^*Tgfbr2*^f/f^ mice (*n*=6–11). (**e**) Foxp3 expression in CD4^+^ T cells from 6-week-old *Foxp3*^Cre^ and *Foxp3*^Cre^*Tgfbr2*^f/f^ mice (*n*=6–11). (**f**) Suppression of proliferation of T_n_ cells labelled with CFSE by different ratios of CD4^+^YFP^+^ T_reg_ cells from *Foxp3*^Cre^ and *Foxp3*^Cre^*Tgfbr2*^f/f^ mice. The experiment was repeated three times. (**g**) Some genes differentially expressed between CD4^+^YFP^+^ T_reg_ cells from *Foxp3*^Cre^ and *Foxp3*^Cre^*Tgfbr2*^f/f^ T_reg_ cells determined by transcriptional profiling. Fold difference means fold change of gene expression levels in T_reg_ cells from *Foxp3*^Cre^*Tgfbr2*^f/f^ mice compared with that from *Foxp3*^Cre^ mice. (**h**,**i**) Expression of indicated proteins on splenic T_reg_ cells from *Foxp3*^Cre^ and *Foxp3*^Cre^*Tgfbr2*^f/f^ mice (*n*=3). (**j**) Suppression of proliferation of T_n_ cells labelled with CFSE by different ratios of CD4^+^YFP^+^ T_reg_ cells from *Foxp3*^Cre^ and *Foxp3*^Cre^*Lkb1*^f/f^ mice that were transduced with retrovirus carrying TGF-βR2 or control vector. (**k**) Foxp3 expression in CD4^+^YFP^+^ T_reg_ cells doubly sorted from *Foxp3*^Cre^ and *Foxp3*^Cre^*Tgfbr2*^f/f^ mice and transferred together with T_n_ cells into *Rag1*^−/−^ mice for 3 weeks. Two-way analysis of variance (ANOVA) was used for statistical analyses in (**a**,**b**,**c,d**,**e**,**i**) (*P<0.05, **P<0.01); error bars represent s.d.; all data are representative of at least two independent experiments.

TGF-β signalling plays crucial roles in T_reg_ cell generation and function^47^. Transcriptional profiling analysis showed that Lkb1-deficient T_reg_ cells express lower levels of *Tgfbr1* and *Tgfbr2* mRNA that was confirmed by real-time PCR and western blot (Fig. 8a and Supplementary Figs 7a and 8s,t). Hence, we proposed that this pathway might be defective in Lkb1-deficient T_reg_ cells. Indeed, Smad2 (Ser 456/457)/Smad3 (Ser 423/425) phosphorylation was decreased (Fig. 8b), and Smad-DNA binding assay indicated that Smad transcriptional activity was impaired in Lkb1-deficient T_reg_ cells (Fig. 8c). To determine whether impaired TGF-β signalling contributed to the impairment of Lkb1-deficient T_reg_ cells, we conducted loss-of-function experiments by generating a mouse line with TGF-βR2 specifically deleted in T_reg_ cells (*Foxp3*^Cre^*Tgfbr2*^f/f^, Supplementary Figs 7b and 8u,v) that displayed decreased Smad activation (Supplementary Fig. 7c). Although *Foxp3*^Cre^*Tgfbr2*^f/f^ mice did not show overt autoimmune disease, increased percentages of CD44^high^CD62L^low^ effector/memory T cells (Fig. 8d) were observed despite a slightly increased T_reg_ cell frequency among CD4^+^ T cells (Fig. 8e), indicating the functional impairment of TGF-βR2-deficient T_reg_ cells. Consistently, TGF-βR2-deficient T_reg_ cells displayed impaired suppressive capacity *in vitro* (Fig. 8f). To determine the global effects of defective TGF-β signalling on gene transcription, we conducted transcriptional profiling and found that TGF-βR2 deficiency led to the decreased expression of many genes important for T_reg_ cell suppressor function (Supplementary Data 4). Remarkably, TGF-βR2 deficiency recapitulated part of the suppressor gene expression alterations in Lkb1-deficient T_reg_ cells, including *Nt5e*, *Ccr6*, *Nrp1*, *Itgae* and *Il1rl1* (Fig. 8g and Supplementary Data 4), that was also confirmed at protein levels (Fig. 8h,i). However, the Lkb1-regulated *Fgl2*, *Il10*, *Tfpi*, *Entpd1* and *Tnfsf8* transcripts were not altered in TGF-βR2-deficient T_reg_ cells, suggesting that Lkb1 also utilize TGF-β-independent mechanisms to promote T_reg_ cell function. To further determine whether lower expression of TGF-βR2 was the causative reason for impaired suppression function of Lkb1-deficient T_reg_ cells, we generated T_reg_ cells (from *Foxp3*^Cre^ and *Foxp3*^Cre^*Lkb1*^f/f^ mice) transduced with retrovirus carrying TGF-βR2 complementary DNA (cDNA). TGF-βR2 was successfully expressed in RFP^+^YFP^+^ T_reg_ cells (Supplementary Fig. 7c). Overexpression of TGF-βR2 could partially rescue the expression of suppressor genes and suppression function of Lkb1-deficient T_reg_ cells (Fig. 8j and Supplementary Fig. 7d,e). Together, these results indicate that Lkb1 implements T_reg_ cell function partially through promoting TGF-β signalling. TGF-βR2-deficient T_reg_ cells did not lose stability when co-transferred with T_n_ cells into *Rag1*^−/−^ mice (Fig. 8k), suggesting that the decreased expression of TGF-β receptors does not account for the instability of Lkb1-deficient T_reg_ cells.

## Discussion

Here, we demonstrate that mice with T_reg_ cell-specific deletion of Lkb1 develop a fatal, early-onset, autoimmune disease with moribund at ∼35 days of age. Such a fulminant disease is comparable to that observed in mice devoid of T_reg_ cells or deficient in *Foxp3*. Similar devastating diseases have also been reported to be present in mice with selective deficiency of Foxo1 (ref. 48) or Raptor, the rapamycin sensitive partner of mTORC1 (mammalian target of rapamycin complex 11)^26^ in T_reg_ cells, due to impaired T_reg_ cell function. Intriguingly, Lkb1-deficient T_reg_ cells lost their linage-specific features and exhibited severe impairment in the maintenance of Foxp3 expression and suppressor function that may underlie the extremely severe autoimmunity in *Foxp3*^Cre^*Lkb1*^f/f^ mice. Although Lkb1 expression is not restricted to T_reg_ cells, T_reg_ cells substantially upregulate Lkb1 protein expression upon TCR stimulation. Given that TCR signalling is essential for T_reg_ cell homeostasis and function, TCR-induced upregulation of Lkb1 protein may represent a critical feed-forward loop to stabilize T_reg_ cell lineage identity.

It is generally recognized that Foxp3 expression is highly stable in T_reg_ cells. Whether a small proportion of T_reg_ cells can lose Foxp3 expression in certain conditions is a controversial topic^6^. Some studies showed that T_reg_ cells could lose Foxp3 expression and become ‘ex-T_reg_’ cells under certain conditions^25^, while others argued that the so-called ‘ex-T_reg_’ cells just might be the conventional T cells that transiently express Foxp3, namely poorly committed T_reg_ cells^27^. Here, we showed that deficiency of Lkb1 caused very severe defects in T_reg_ cell stability. More than 70% of T_reg_ cells lost stability in *Foxp3*^Cre^*Lkb1*^f/f^*Rosa26*^YFP^ mice. In addition, the majority of the Lkb1-deficientT_reg_ cells lost Foxp3 expression after being transferred into *Rag1*^−/−^mice, indicating that Lkb1-deficient mature T_reg_ cells continuously lost Foxp3 expression, rather than that a subset of Lkb1-deficient T cells only transiently expressed Foxp3 (poorly committed T_reg_ cells). Such a severe defect was rarely reported in literatures, placing Lkb1 as a particularly important maintainer of Foxp3 stability. The demethylated status of *Foxp3* CNS2 is critically involved in the maintenance of stable Foxp3 expression in T_reg_ cells^8^,^9^,^10^,^28^,^29^,^30^,^31^,^32^. Substantial methylation of CNS2 but not promoter was observed in Lkb1-deficient T_reg_ cells. Treatment of IL-12 further enhanced the methylation of CNS2 and caused the loss of Foxp3 expression in Lkb1-deficient but not wild-type T_reg_ cells. Moreover, the DNA methyltransferase inhibitor 5-aza-dC reduced the methylation of CNS2 and rescued the instability of Lkb1-deficient T_reg_ cells. These results suggest that Lkb1 stabilizes Foxp3 expression by building up the demethylated status of CNS2. It has been recently reported that deletion of CNS2 caused cell-division-dependent loss of Foxp3 expression^8^,^10^. In contrast, both divided and nondivided Lkb1-deficient T_reg_ cells lost Foxp3 expression, suggesting that Lkb1 might also promote Foxp3 expression by CNS2-independent mechanisms, which need future exploration.

The role of STAT4 in T_reg_ cells is not well defined. Here we showed that deficiency of Lkb1 in T_reg_ cells resulted in markedly more IL-12-induced STAT4 activation and STAT4 binding to CNS2. STAT4 associated with Dnmt1 and DNA methyltransferase chemical inhibitor rescued the instability of Lkb1-deficient T_reg_ cells in response to IL-12. Thus, Lkb1 suppresses IL-12/STAT4 activation to stabilize Foxp3 expression in T_reg_ cells. We further showed that NF-κB signalling is activated in Lkb1-deficient T_reg_ cells, that increased *Il12rb2* and *Stat4* mRNA expression and STAT4 activation. However, inhibition of NF-κB signalling can only partially rescue the instability of Lkb1-deficient T_reg_ cells, implicating that NF-κB-independent signalling mechanisms might also contribute to the unstable Foxp3 expression in Lkb1-deficient T_reg_ cells.

Independent of maintaining Foxp3 expression, Lkb1 also promotes T_reg_ cell-suppressive capacity. Loss of Lkb1 in T_reg_ cells results in the moderate downregulation of a number of immunosuppressive genes (*Fgl2*, *Tfpi*, *Cybb*, *Entpd1*, *Nt5e*, *Nrp1*, *Itgae*, *Il18*, *Il10*, *Ccr6*, *Il1rl1*), suggesting that Lkb1 implements T_reg_ cell function through broadly promoting T_reg_ suppressor gene expression program. Lkb1 has been previously shown to positively or negatively regulate TGF-β/Smad signalling in a cell type-dependent manner^49^,^50^, but the precise mechanism is not fully elucidated. Here, we found the effect of Lkb1 on promoting T_reg_ suppressor function was partially dependent on its augmentation of TGF-β signalling. TGF-β pathway is important for T_reg_ cell development in the thymus and periphery^47^,^51^,^52^, but whether and how it affects T_reg_ cell function remain poorly understood. Our results demonstrate that TGF-βR2-deficient T_reg_ cells have impaired suppressive capacity associated with decreased expression of a number of genes associated with immune suppression (*Nt5e*, *Ccr6*, *Nrp1*, *Itgae* and *Il1rl1*), partially recapturing the suppressor gene expression alterations in Lkb1-deficient T_reg_ cells. Furthermore, overexpression of TGF-βR2 partially rescued the expression of certain suppressor genes and the suppressor function of Lkb1-deficient T_reg_ cells, supporting that impaired TGF-β signalling contributed to the impaired suppressor function of Lkb1-deficient T_reg_ cells. However, numerous Lkb1-regulated genes, including *Fgl2*, *Tfpi*, *Cybb*, *Entpd1* and *Il18*, were not changed in TGF-βR2-deficient T_reg_ cells, suggesting that TGF-β-independent mechanisms were also applied by Lkb1 to promote T_reg_ cell suppressor function.

AMPK-induced fatty acid oxidation is a critical metabolic feature of T_reg_ cells^53^. To our surprise, AMPK activation is not dramatically altered in Lkb1-deficient T_reg_ cells, and T_reg_ cell-specific deletion of both AMPK1α and AMPK2α does not cause T_reg_ cell abonrmality and immune disturbance, suggesting that Lkb1 does not control T_reg_ cells by promoting AMPK activity in steady-state conditions.

Lkb1 has been previously shown to promote thymocytes development, maintain survival and proliferation of peripheral T cells and restrain peripheral conventional T-cell activation and proinflammatory cytokine production^15^,^16^,^54^ (we got similar results in certain experiments after inducing Lkb1 deletion in conventional T cells from *ERT2*^Cre^*Lkb1*^f/f^ mice, Supplementary Fig. 7f,g). Here, we find that Lkb1 maintains the lineage identity of T_reg_ cells through mechanisms involving the regulation of NF-κB/STAT4 and TGF-β signallings (Fig. 8). NF-κB/STAT4 and TGF-β signallings are separately controlled by Lkb1 (Supplementary Fig. 7h–j), suggesting a function of Lkb1 in setting up the optimal activation thresholds of multiple intracellular signallings in T_reg_ cells. Furthermore, the upregulation of Lkb1 protein by TCR signalling in T_reg_ but not in conventional T cells indicates a specific regulation of Lkb1 expression required for preserving the T_reg_ cell lineage. Future studies will determine whether and how the environmental cues change Lkb1 in T_reg_ cells to affect their stability/function under physiological and pathological conditions, and whether Lkb1 can be targeted to treat T_reg_-cell-related immune diseases.

## Methods

### Mice

All animals were maintained in specific pathogen-free barrier facilities and were used in accordance with protocols approved by the institutional animal care and user committee at the Institute of Hematology, Chinese Academy of Medical Sciences. C57BL/6, CD45.1^+^, *Lkb1*^f/f^, *AMPKα1*^f/f^, *AMPKα2*^f/f^, *Tgfbr2*^f/f^, *ERT2*^Cre^, *Foxp3*^YFP-Cre^ (*Foxp3*^Cre^), *Rosa26*^YFP^ and *Rag1*^−/−^ mice were purchased from Jackson Laboratories. All mice had been backcrossed with C57BL/6 mice for at least 7 generations. *Lkb1*^f/f^, *AMPKα1*^f/f^, *AMPKα2*^f/f^, *Tgfbr2*^f/f^ mice were crossed with *Foxp3*^Cre^ mice to generate *Foxp3*^Cre^*Lkb1*^f/f^, *Foxp3*^Cre^*AMPKα1*^f/f^*AMPKα2*^f/f^ and *Foxp3*^Cre^*Tgfbr2*^f/f^ mice, respectively. *Foxp3*^Cre/+^*Lkb1*^f/f^ mice were crossed with *Rosa26*^YFP^ mice to generate *Foxp3*^Cre^*Lkb1*^f/f^*Rosa26*^YFP^ mice. *Lkb1*^f/f^ mice were crossed with *ERT2*^Cre^ and *Rosa26*^YFP^ mice to generate *ERT2*^Cre^*Lkb1*^f/f^*Rosa26*^YFP^ mice. Sample size for various animal experiments was chosen based on prior data generated in the laboratory, and no mice were excluded from experiments. The details of mice age and sex are provided in the figure legends.

### Cell purification and flow cytometry

Single-cell suspensions were prepared from spleen and peripheral lymph nodes for staining or cell purification. CD4^+^ T cells and CD11c^+^ DCs were purified with Dynabeads Untouched Mouse CD4 Cells Kits (Invitrogen, 11415D) or CD11c MicroBeads (Miltenyi Biotec, 130-108-338), respectively. Indicated T-cell populations were sorted from purified CD4^+^ T cells with FACSAria III (BD Biosciences), and the sorted populations were >98% pure unless otherwise specified. Flow cytometry of cell surface molecules was performed as previously described^55^, and the antibody (Supplementary Data 5) was diluted as the instructions of the manufacturers suggested. Intracellular staining of Foxp3, cytokines (spleen cells were stimulated with phorbol myristate acetate (50 ng ml^−1^) and ionomycin (500 ng ml^−1^) for 4 h before analysis of cytokine expression in indicated populations) and other proteins were performed with Foxp3 staining kits (eBioscience, 00-5523). Intracellular staining of phosphorylated proteins was performed on cells fixed with methanol and permeabilized with Triton X-100. The antibodies were obtained from eBioscience, Biolegend, BD Biosciences, Cell Signalling Technology, R&D Systems and Invitrogen, and listed in Supplementary Data 5. Flow cytometry data were acquired on LSR II, LSRFortessa (BD Biosciences) or FACSCanton II (BD Biosciences) and analysed with Flowjo software (Tree Star).

### Cell culture

Sorted CD4^+^YFP^+^ T_reg_ cells from *Foxp3*^Cre^*Lkb1*^f/f^ and *Foxp3*^Cre^ mice were labelled with carboxyfluorescein succinimidyl ester (CFSE) Cell Proliferation Kits (Invitrogen, C34554). CFSE-labelled T_reg_ cells were co-cultured with CD11c^+^ cells purified from CD45.1^+^ mice, supplemented with indicated combinations of cytokines of recombinant murine IL-2, IL-12, IL-6 and IL-4 (all 100 ng ml^−1^, PeproTech), NF-κB inhibitors sodium 4-aminosalicylate (4-ASA) or pyrrolidinedithiocarbamic acid (PDTC) or DNA methyltransferase inhibitor 5-aza-dC)in Dulbecco’s modified Eagle’s medium supplemented with 10% fetal bovine serum (Gibco) for 4 days. For inducible Lkb1 deletion, T_reg_ cells were respectively sorted from *ERT2*^Cre^*Lkb1*^f/f^*Rosa26*^YFP^ and *ERT2*^Cre^*Rosa26*^YFP^ mice and cultured for 5 days as described above, with the addition of 4-hydroxytamoxifen (1 μM, Sigma) in the culture. In other experiments, sorted T_reg_ cells were incubated in plates precoated with anti-CD3 (2 μg ml^−1^; 145-2C11; eBioscience) and anti-CD28 (2 μg ml^−1^; 45.21; eBioscience) in the presence of IL-2 (100 ng ml^−1^) for the indicated days. CFSE profiles and Foxp3 expressions were examined by flow cytometry after culturing.

### *In vivo* T_reg_ cell maintenance

CD4^+^CD25^+^YFP^+^ T_reg_ cells were doubly sorted (>99.5% pure) from 3-week-old CD45.1^−^ CD45.2^+^ *Foxp3*^Cre^*Lkb1*^f/f^ and CD45.1^+^CD45.2^+^*Foxp3*^Cre^ mice, and 2 × 10^5^ of each type of cells were mixed with 4 × 10^5^ CD4^+^CD25^−^CD44^low^CD62L^high^ T_n_ cells sorted fromCD45.1^+^ CD45.2^−^ mice and transferred into the *Rag1*^−/−^ mice by intraperitoneal injection. The recipient mice were analysed 3 weeks after transfer.

### *In vitro* T_reg_ cell suppression

CD4^+^CD25^−^CD44^low^CD62L^high^ T_n_ cells sorted from CD45.1^+^ mice were labelled with CFSE and used as responder cells (T_resp_). T_resp_ cells (5 × 10^4^) were cultured for 72 h with DCs (1 × 10^5^) and soluble anti-CD3 (2 μg ml^−1^) in the presence or absence of the indicated numbers of CD4^+^YFP^+^ T_reg_ cells sorted from *Foxp3*^Cre^*Lkb1*^f/f^ or *Foxp3*^Cre^ mice.

### *Foxp3* gene methylation assay

Genomic DNA from sorted cells was bisulfite converted with EZ DNA Methylation-Direct kits according to the manufacturer’s protocol (Epigentek, P-1026-050). Methylation-specific PCR primers (listed in Supplementary Data 2) were used for amplification of the promoter and intron 1 of *Foxp3* (corresponding to *Foxp3* conserved noncoding sequence 2). PCR products were subcloned into pGEM-T Easy vectors (Promega) and sequenced.

### Western blot

CD4^+^YFP^+^ T_reg_ cells and CD4^+^YFP^−^ conventional T cells were sorted from *Foxp3*^Cre^ and *Foxp3*^Cre^*Lkb1*^f/f^ mice and treated as indicated, and then cells were lysed and western blot was carried out as previously described^55^ with antibodies to Lkb1 (D60C5, Cell Signalling Technology), phosphorylated AMPK (40H9, Cell Signalling Technology), IKKβ (2C8, Cell Signalling Technology), phosphorylated IKKα/β (16A6, Cell Signalling Technology), phosphorylated IκBα (14D4, Cell Signalling Technology), phospho-NF-κB p65 (93H1, Cell Signalling Technology), NF-κB p65 (D14E12, Cell Signalling Technology), STAT4 (C46B10, Cell Signalling Technology), AMPKα (D63G4, Cell Signalling Technology), phospho-Acetyl-CoA Carboxylase (D7D11, Cell Signalling Technology), phospho-STAT4 (Abcam), TGF-βR2 (Abcam) and GAPDH (D16H11, Cell Signalling Technology).

### Microarray and quantitative real-time PCR

CD4^+^YFP^+^ T_reg_ cells were sorted from spleen and lymph nodes of mice for RNA extraction with Trizol reagent (Invitrogen). Total RNA was reverse transcribed, amplified, labelled and hybridized to Mouse Genome 2.0 arrays (Affymetrix). Microarray data sets were analysed with Agilent Genespring GS 11 software. RNA of different samples was obtained in the same manner as in microarray analysis, and real-time PCR was performed with SYBR Green PCR Master Mix (ABI) as previously described^55^. The sequences of the primer pairs used are listed in Supplementary Data 2.

### DNA binding ELISA

TransAM Flexi kits (Active Motif, 40098) were used to test the activity of Smad transcription factors according to the manufacturer’s protocol. Briefly, nuclear extract from stimulated T_reg_ cells sorted from *Foxp3*^Cre^ and *Foxp3*^Cre^*Lkb1*^f/f^ mice were mixed with biotinylated oligos (listed in Supplementary Data 2) recognized by transcription factors of interest, and incubated in a streptavidin-coated plate. After washing, primary antibodies specific for the bound transcription factor and horseradish peroxidase-conjugated secondary antibodies were subsequently added, and transcription factor binding activities were quantified using a microplate reader (Synergy H4, BioTek).

### Co-immunoprecipitation

Sorted wild-type CD4^+^YFP^+^ T_reg_ cells were expanded in plates coated with anti-CD3 (2 μg ml^−1^) and anti-CD28 (2 μg ml^−1^) in the presence of IL-2 (100 ng ml^−1^) for 5 days and collected. Cell lysis was prepared using Co-immunoprecipitation kits according to the manufacturer’s protocol (Sigma, FLAGITP1), and incubated with antibodies against Dnmt1 (60B1220.1, Abcam), Dnmt3a (Abcam) and protein G agarose (Cell Signalling Technology). Immunoprecipitated proteins were detected as described above with antibodies against STAT4 (C46B10, Cell Signalling Technology) and STAT5 (Cell Signalling Technology).

### Chromatin immunoprecipitation

ChIP assays were performed using the ChIP kits (Active Motif, 53040) according to the manufacturer’s protocol. Precipitated DNA and input DNA were assessed by real-time PCR using primers listed in Supplementary Data 2.

### Retroviral transduction

TGF-βR2 cDNA was subcloned into the retroviral vector pMYs-IRES-RFP. Retroviruses were produced by transfection of the Plat-E cells with polyethylenimin. T_reg_ cells (YFP^+^) were cultured in plates coated with anti-CD3 and anti-CD28 transduced in virus-containing media supplemented with polybrene by centrifuging for 1 h at 500 RCF. After 24 h, cells were expanded and then RFP^+^ YFP^+^ cells were sorted before further analysis. The Plat-E cell line was provided by the Cell Resource Center, Institute of Hematology and Hospital of Blood Diseases, Chinese Academy of Medical Sciences/Peking Union Medical College. The Cell Resource Center confirmed the species origin with PCR and checked free of mycoplasma contamination by PCR and culture.

### Histopathology

Skins, lungs, livers and stomachs were removed from 3–4-week-old mice. Samples were formalin fixed, paraffin embedded and stained with haematoxylin and eosin before tissue histology. Photomicrographs were taken at × 10 or × 20 magnifications.

### Statistics

An unpaired two-tailed Student’s *t*-test (for two group comparisons) or a two-way analysis of variance (for more than two group comparisons) were performed using Prism (GraphPad) to calculate statistical significance of the difference in mean values and *P* values. A *P* value of <0.05 was considered statistically significant. *P<0.05; **P<0.01. No specific randomization or blinding protocols were used.

### Data availability

The data that support the findings of this study are available from the corresponding author on reasonable request. The microarray data have been deposited in the Gene Expression Omnibus under accession GSE97840.

## 

## Additional information

**How to cite this article:** Wu, D. *et al*. Lkb1 maintains T_reg_ cell lineage identity. *Nat. Commun.* **8**, 15876 doi: 10.1038/ncomms15876 (2017).

**Publisher’s note:** Springer Nature remains neutral with regard to jurisdictional claims in published maps and institutional affiliations.

## Supplementary Material

Supplementary Information

Supplementary Data 1

Supplementary Data 2

Supplementary Data 3

Supplementary Data 4

Supplementary Data 5
