# Supplementary material for: Lkb1 maintains Treg cell lineage identity
Source: Nat Commun. 2017 Jun 16;8:15876. doi: 10.1038/ncomms15876 (PMC5481770; doi:10.1038/ncomms15876)
Supplement: Supplementary Information [file ncomms15876-s1.pdf]

Type of file: pdf
Size of file: 0 KB
Title of file for HTML: Supplementary Information
Description: Supplementary Figures

Type of file: xlsx
Size of file: 0 KB
Title of file for HTML: Supplementary Data 1
Description: Schematic description of transcription factor binding motifs in Foxp3, Stat4 and Il12rb2 locus.

Type of file: xlsx
Size of file: 0 KB
Title of file for HTML: Supplementary Data 2
Description: Primers and peptide sequences.

Type of file: xlsx
Size of file: 0 KB
Title of file for HTML: Supplementary Data 3
Description: Gene expression alterations in Lkb1-deficient Treg cells 1.5 fold change.

Type of file: xlsx
Size of file: 0 KB
Title of file for HTML: Supplementary Data 4
Description:  Gene expression alterations in TGF-βR2-deficient Treg cells 1.5 fold change.

Type of file: xlsx
Size of file: 0 KB
Title of file for HTML: Supplementary Data 5
Description: Antibodies for flow cytometry.

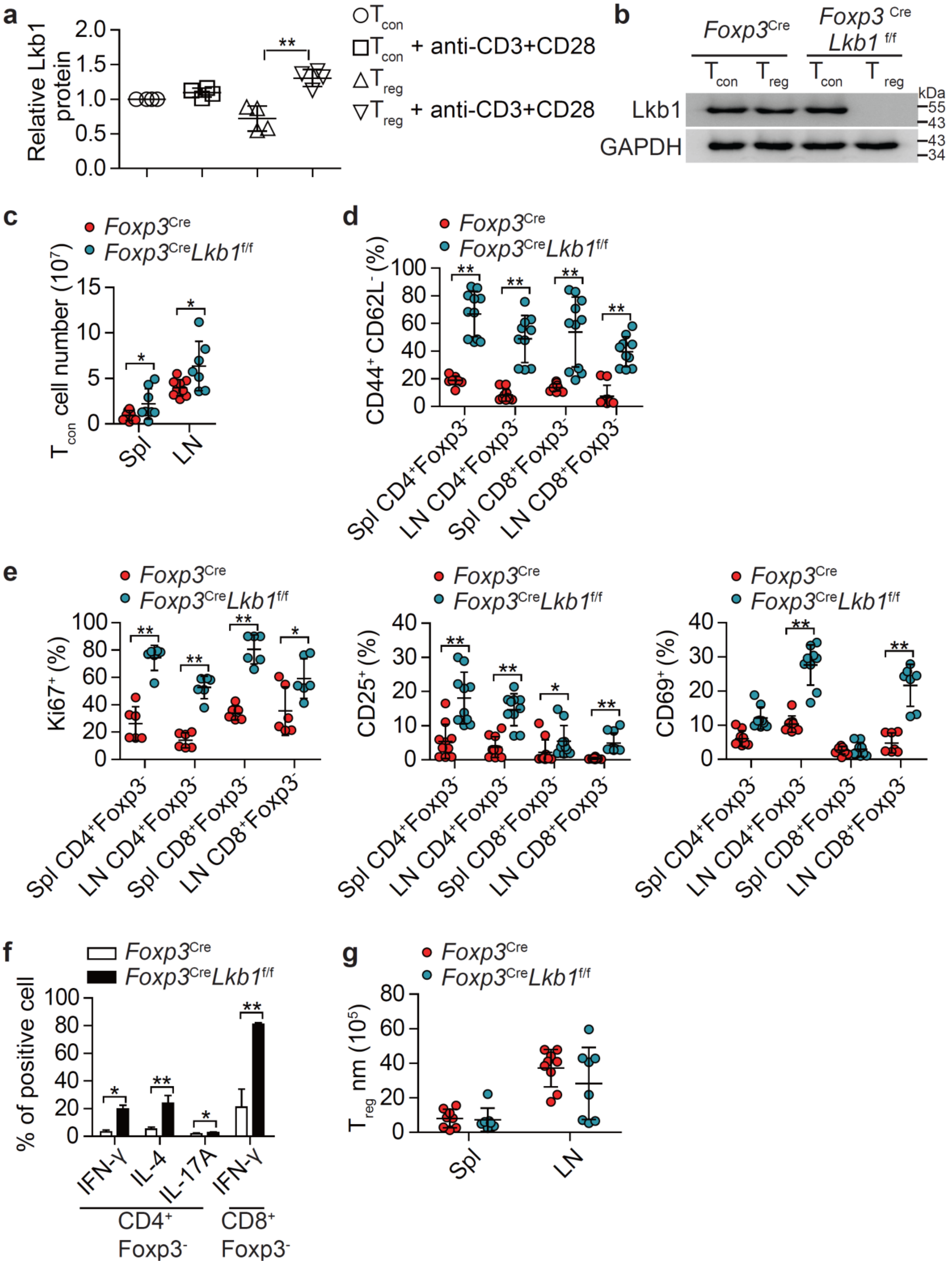


**Supplementary Figure 1. $T_{reg}$ cell-specific deletion of Lkb1 causes T cell autoimmunity.(a)** Relative expression of Lkb1 proteins in $CD4^{+}YFP^{-}$conventional T ($T_{con}$) cells and $CD4^{+}YFP^{+}T_{reg}$ cells un-treated or stimulated in plates coated with anti-CD3 and anti-CD28 in the presence of IL-2 for 24 h determined by western blot, and calculated by densitometry after normalization with GAPDH (n=4). (**b**)Lkb1 protein was depleted in $CD4^{+}YFP^{+}T_{reg}$ cells from 2-week-old *Foxp3*$^{Cre}$*Lkb1*$^{f/f}$ mice, determined by western blot. (**c**)$T_{con}$ cell numbers in spleen and lymph nodes of *Foxp3*$^{Cre}$ and *Foxp3*$^{Cre}$*Lkb1*$^{f/f}$ mice (n=7-9).(**d**) Percentages of $CD44^{high}CD62L^{low}$ effector/memory

cells among $CD4^+Foxp3^-$ and $CD8^+Foxp3^-$ T cells from spleen and lymph nodes of *Foxp3*$^{Cre}$ and *Foxp3*$^{Cre}$*Lkb1*$^{f/f}$ mice (n=6-11).(**e**) Percentages of cells positive for Ki67, CD25 or CD69 among $CD4^+Foxp3^-$ and $CD8^+Foxp3^-$ T cells in spleen and lymph nodes from *Foxp3*$^{Cre}$ and *Foxp3*$^{Cre}$*Lkb1*$^{f/f}$ mice (n=6-10).(**f**) Cytokine production in PMA and ionomycin-stimulated (4 h) splenic $CD4^+Foxp3^-$ and $CD8^+Foxp3^-$ T cells from *Foxp3*$^{Cre}$ and *Foxp3*$^{Cre}$*Lkb1*$^{f/f}$ mice (n=4). All mice analyzed were 28-30-day-old, unless otherwise specified. (**g**) Absolute numbers of $CD4^+Foxp3^+$ $T_{reg}$ cells in the spleen and lymph nodes from 28-30-day-old *Foxp3*$^{Cre}$ and *Foxp3*$^{Cre}$*Lkb1*$^{f/f}$ mice (n=6-8). Two-way ANOVA was used for statistical analyses in **a**, **c**, **d, e**, **f**, and **g** (**P*<0.05, ***P*<0.01); error bars represent s.d.; all data are representative of at least two independent experiments.

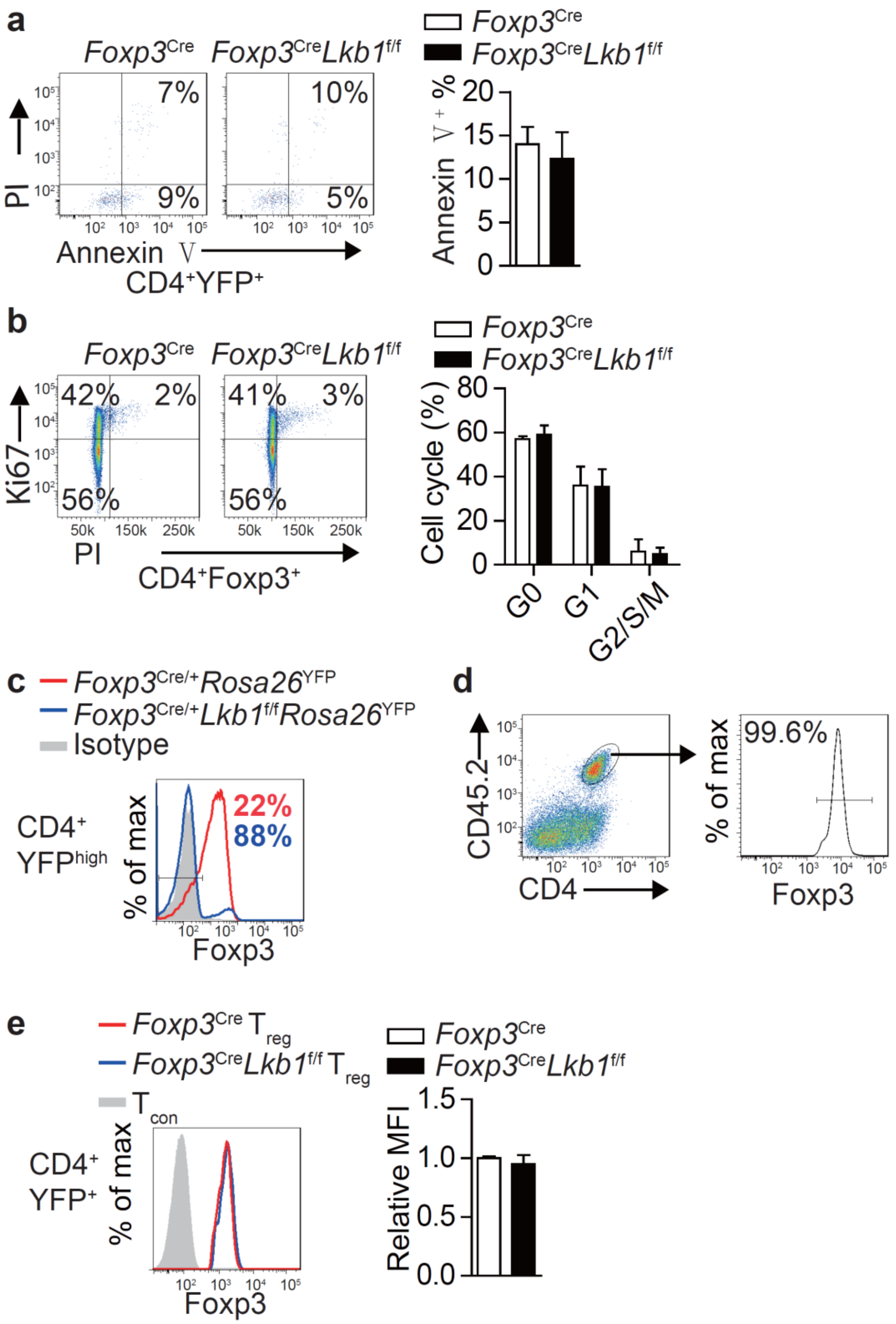


**Supplementary Figure 2. Homeostasis of Lkb1-deficient T$_{reg}$ cells in vivo.** (**a**)Annexin V and PI staining of splenic CD4$^{+}$YFP$^{+}$ T$_{reg}$ cells from *Foxp3*$^{Cre}$ and *Foxp3*$^{Cre}$*Lkb1*$^{f/f}$ mice (n=3). (**b**) Ki67 and PI staining of splenic CD4$^{+}$Foxp3$^{+}$ T$_{reg}$ cells from *Foxp3*$^{Cre}$ and *Foxp3*$^{Cre}$*Lkb1*$^{f/f}$ mice (n=3). (**c**) Foxp3 expression in CD4$^{+}$*Rosa26*-YFP$^{high}$ cells from the heterozygous female *Foxp3*$^{Cre/+}$*Rosa26*$^{YFP}$ and *Foxp3*$^{Cre/+}$*Lkb1*$^{f/f}$*Rosa26*$^{YFP}$ mice.(**d**) The T$_{reg}$ cell purity after sorting was more than 99.5%.(**e**)Foxp3 expression in CD4$^{+}$YFP$^{+}$ T$_{reg}$ cells sorted from *Foxp3*$^{Cre}$ and *Foxp3*$^{Cre}$*Lkb1*$^{f/f}$ mice. Two-way ANOVA was used for statistical analyses in **b,** and unpaired two-tailed Student's t-test was used for statistical analyses in **a** and **e**; error bars represent s.d.; all data are representative of at least two independent experiments.

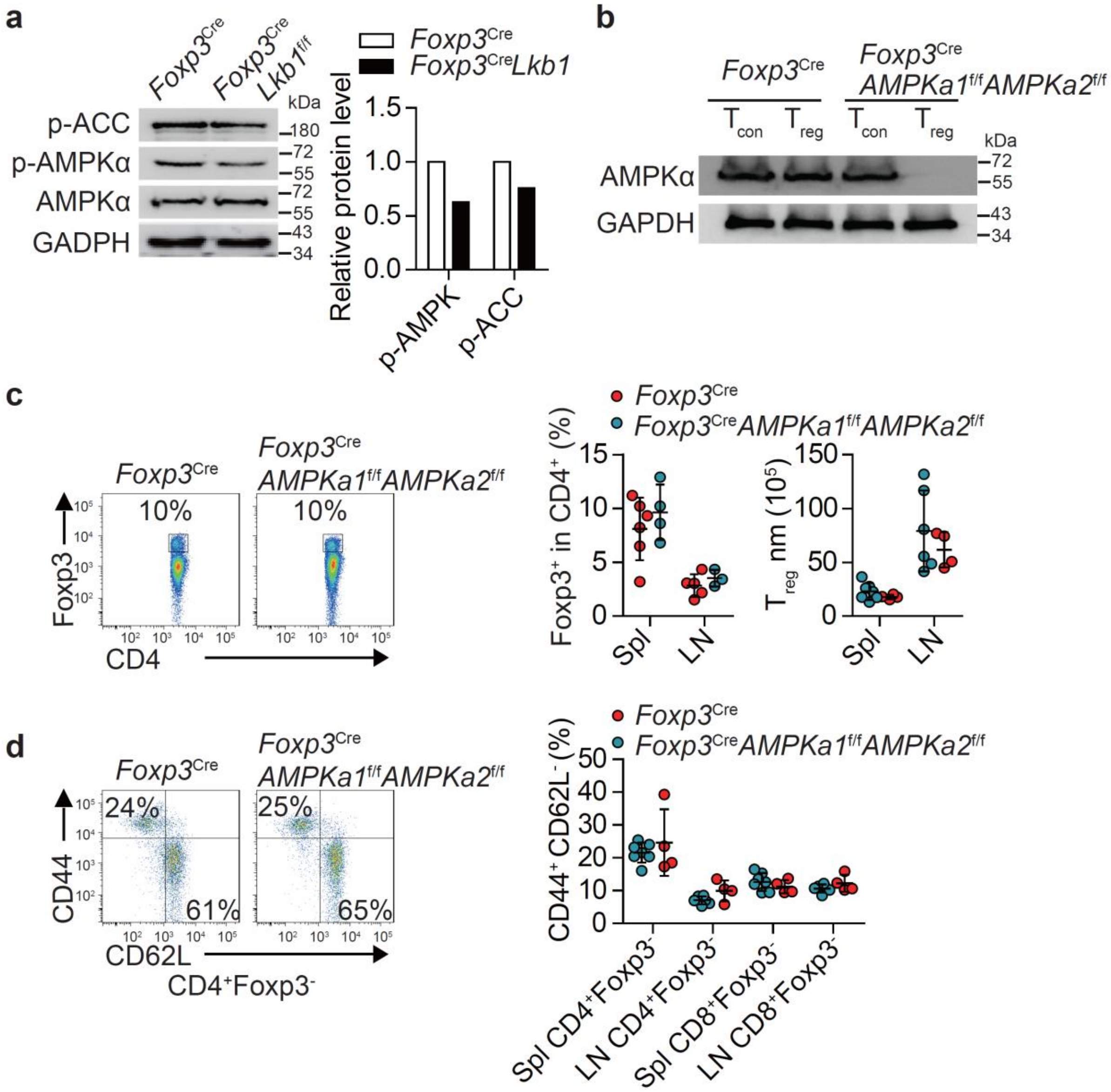


**Supplementary Figure3. Lkb1 functions in $T_{reg}$ cells independent of AMPK.**(**a**) A representative image of phosphorylated acetyl-coa carboxylase (ACC, Ser79), phosphorylated AMPKα (Thr172),and total AMPKαexpression in *Foxp3*$^{Cre}$ and *Foxp3*$^{Cre}$*Lkb1*$^{f/f}$ $T_{reg}$ cells, determined by western blot,and relative protein levels calculated by densitometry after normalization with GAPDH.(**b**) AMPKα protein was depleted in CD4$^{+}$YFP$^{+}$ $T_{reg}$ cells from *Foxp3*$^{Cre}$*Lkb1*$^{f/f}$ mice, determined by western blot. (**c**) Foxp3 expression in CD4$^{+}$ T cells from *Foxp3*$^{Cre}$ and *Foxp3*$^{Cre}$*AMPKα1*$^{f/f}$*AMPKα2*$^{f/f}$ mice (n=4-6). (**d**) CD44$^{high}$CD62L$^{low}$ effector/memory cells among CD4$^{+}$Foxp3$^{-}$ and CD8$^{+}$Foxp3$^{-}$ T cells from spleen and lymph nodes of*Foxp3*$^{Cre}$ and *Foxp3*$^{Cre}$*AMPKα1*$^{f/f}$*AMPKα2*$^{f/f}$ mice (n=4-6). Two-way ANOVA was used for statistical analyses in **c** and **d**; error bars represent s.d.; all data are representative of at least two independent experiments.

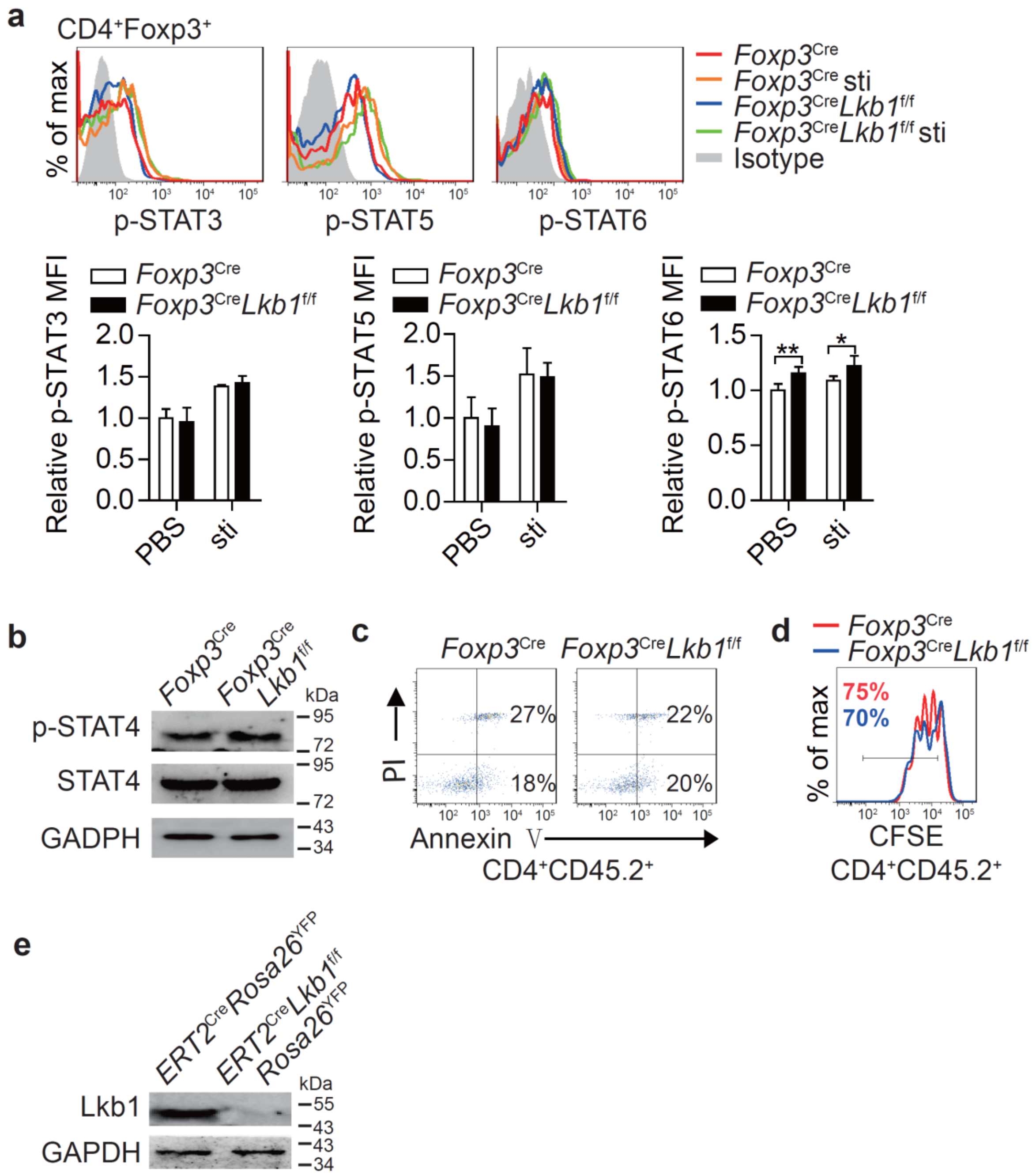


**Supplementary Figure 4. Intracellular signaling alterations in Lkb1-deficient $T_{reg}$ cells.** (**a**) Intracellular phosphorylated STAT3, STAT5 and STAT6 in *Foxp3*$^{Cre}$ and *Foxp3*$^{Cre}$*Lkb1*$^{f/f}$ $T_{reg}$ cells, stimulated with or without IL-6, IL-2, IL-4, respectively (n=3). (**b**)A representative image of phosphorylated STAT4 (Tyr693) and total STAT4 expression in *Foxp3*$^{Cre}$ and *Foxp3*$^{Cre}$*Lkb1*$^{f/f}$ $T_{reg}$ cells, determined by western blot. (**c**) Apoptosis of $T_{reg}$ cells from *Foxp3*$^{Cre}$ and *Foxp3*$^{Cre}$*Lkb1*$^{f/f}$ mice, co-cultured with DCs supplemented with IL-2+IL-12.(**d**) Proliferation of CFSE-labeled $T_{reg}$ cells from *Foxp3*$^{Cre}$ and *Foxp3*$^{Cre}$*Lkb1*$^{f/f}$ mice, co-cultured with DCs supplemented with IL-2+IL-12. (**e**) Lkb1 protein was depleted in *ERT2*$^{Cre}$*Rosa26*$^{YFP}$ and *ERT2*$^{Cre}$*Lkb1*$^{f/f}$*Rosa26*$^{YFP}$$T_{reg}$ cells co-cultured with DCs supplemented with indicated cytokines and 4-hydroxytamoxifen, determined by western blot. Two-way ANOVA was used for statistical analyses in **a** (**P*<0.05, ***P*<0.01); error bars represent s.d.; data represent at least three independent experiments.

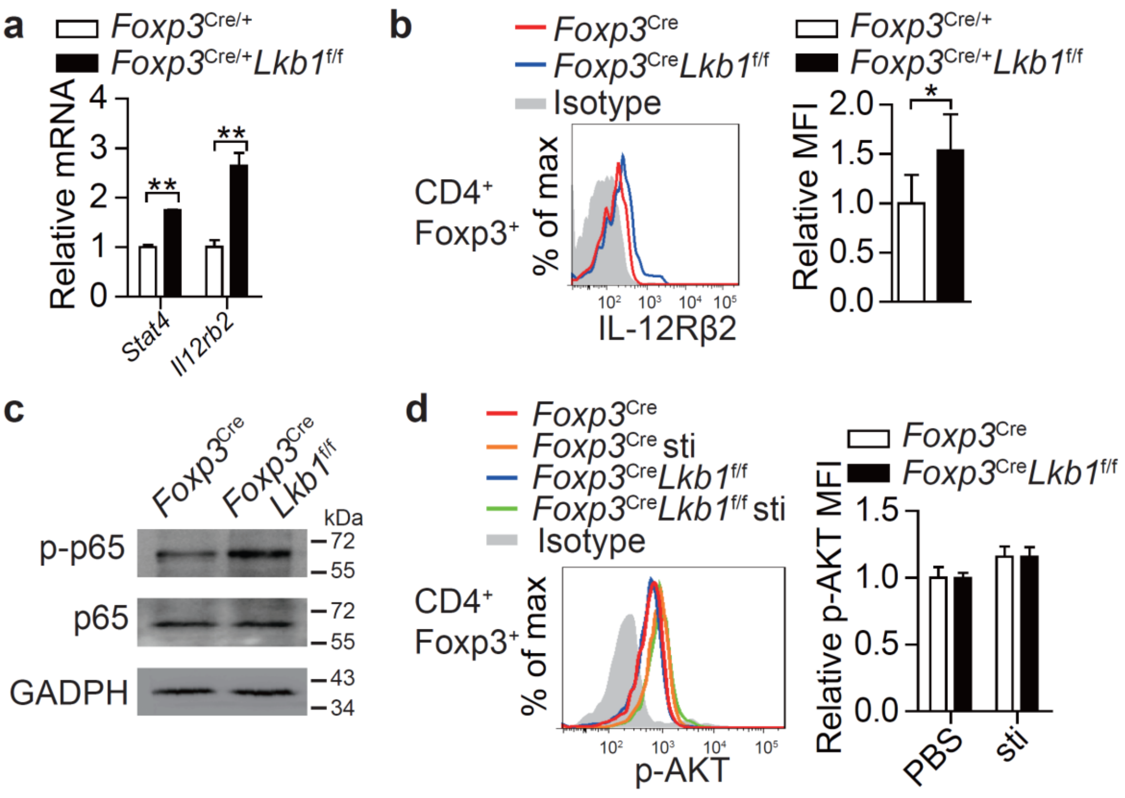


**Supplementary Figure 5. Expression of *Stat4*, *Il12rb2* andphosphorylated p65 was increased in Lkb1-deficient $T_{reg}$ cells.** (**a**) *Stat4* and *Il12rb2* mRNA expression in *Foxp3*$^{Cre}$ and *Foxp3*$^{Cre}$*Lkb1*$^{f/f}$ $T_{reg}$ cells (n=3). (**b**) IL-12Rβ2 expression on *Foxp3*$^{Cre}$ and *Foxp3*$^{Cre}$*Lkb1*$^{f/f}$ $T_{reg}$ cells (n=3). (**c**) A representative image of phosphorylated p65 (Ser536) and total p65 expression in *Foxp3*$^{Cre}$ and *Foxp3*$^{Cre}$*Lkb1*$^{f/f}$ $T_{reg}$ cells, determined by western blot. (**d**) Intracellular phosphorylated AKT in *Foxp3*$^{Cre}$ and *Foxp3*$^{Cre}$*Lkb1*$^{f/f}$ $T_{reg}$ cells with or without IL-2 stimulation (n=3). Two-way ANOVA was used for statistical analyses in **a** and **d,** and unpaired two-tailed Student's t-test was used for statistical analyses in **b** (*$P$<0.05); error bars represent s.d.; data represent at least three independent experiments.

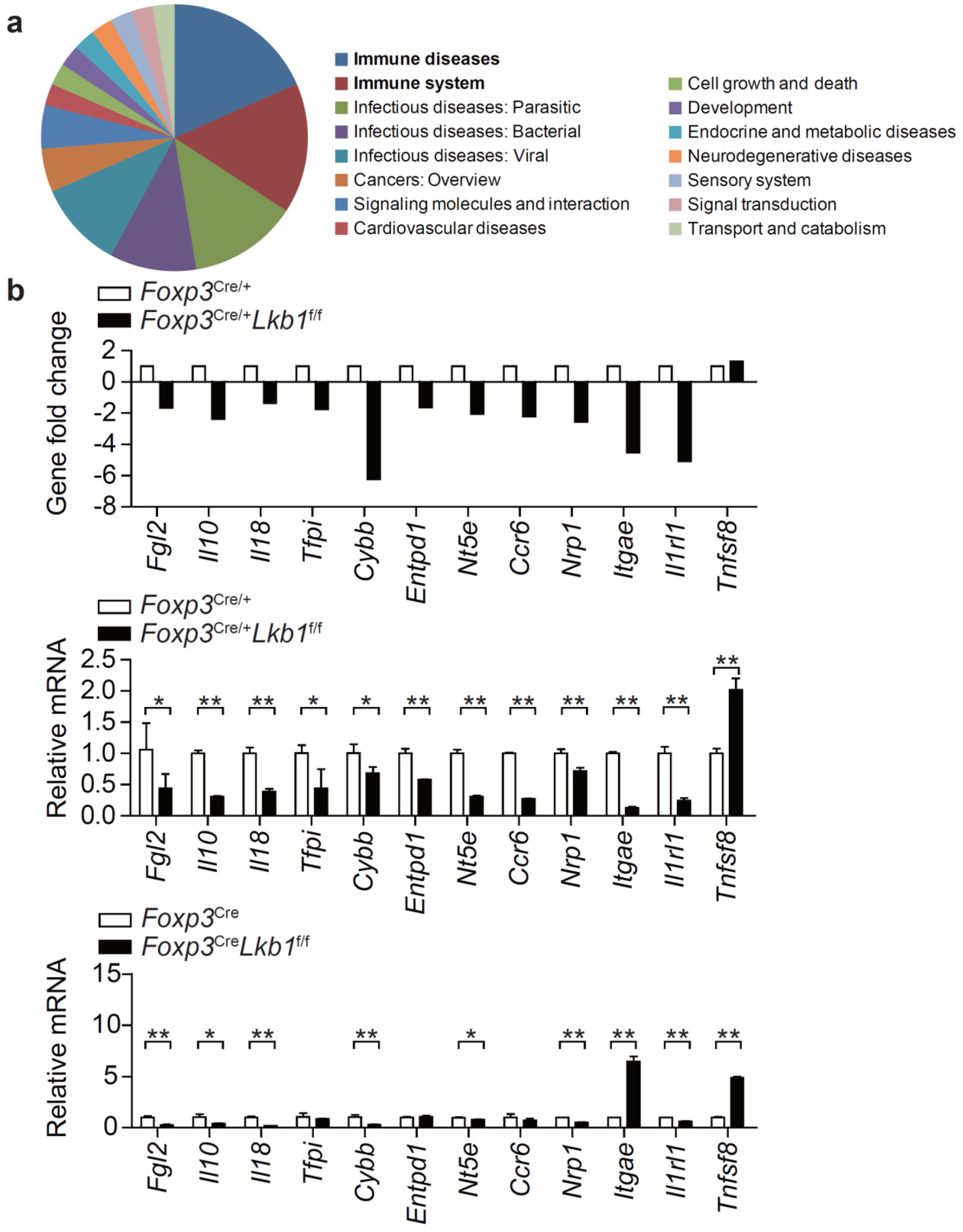


**Supplementary Figure 6. Lkb1 controls the expression of genes critical for $T_{reg}$ cell function.** (**a**) Top enriched KEGG pathways. (**b**) mRNA levels of selected genes by transcriptional profiling and real-time PCR (n=3). *Foxp3*$^{Cre/+}$ versus *Foxp3*$^{Cre/+}$*Lkb1*$^{f/f}$ $T_{reg}$ cells or *Foxp3*$^{Cre}$ versus *Foxp3*$^{Cre}$*Lkb1*$^{f/f}$ $T_{reg}$ cells (n=3). Two-way ANOVA was used for statistical analyses in **b** (**P*<0.05, ***P*<0.01); error bars represent s.d.; data are representative of at least two independent experiments.

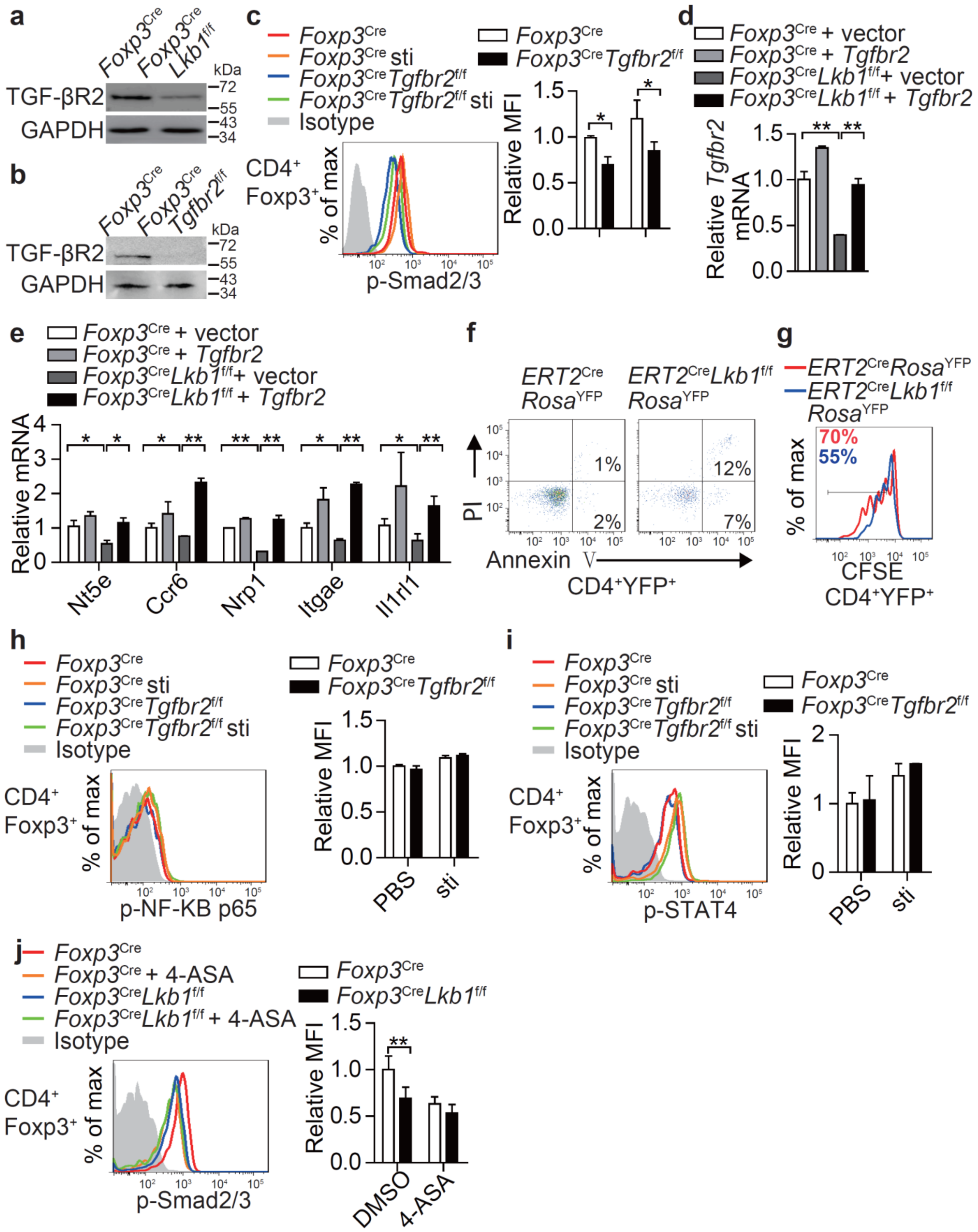


**Supplementary Figure 7. Over-expression of TGF-βR2 could partially rescue the expression of suppressor genes of Lkb1-deficient $T_{reg}$ cells and NF-κB/STAT4 and TGF-β signaling are independent in Lkb1-deficient $T_{reg}$ cells.** (**a**) TGF-βR2 protein was decreased in Lkb1-deficient $T_{reg}$ cells compared with wild-type $T_{reg}$ cells. (**b**) TGF-βR2 protein was depleted in $CD4^+YFP^+$ $T_{reg}$ cells from *Foxp3*$^{Cre}$*TGF-βR2*$^{f/f}$ mice, determined by western blot.(**c**) Intracellular expression of phosphorylated Smad2/3 in $T_{reg}$ cells from *Foxp3*$^{Cre}$ and *Foxp3*$^{Cre}$*Tgfbr2*$^{f/f}$ mice, with or without TGF-β stimulation (n=3). (**d**) TGF-βR2 was successfully expressed in $RFP^+$ $YFP^+$ $T_{reg}$ cells after

transduced with TGF-βR2 cDNA carrying retrovirus. (**e**) Over-expression of TGF-βR2 could partially rescue the expression of suppressor genes of Lkb1-deficient $T_{reg}$ cells. $RFP^+$ $YFP^+$ $T_{reg}$ cells were isolated 48h after transduced with retrovirus, the expression of indicated genes was determined by flow cytometry (n=3). pMYs-IRES-RFP vectorwas used as a control. (**f,g**) $CD4^+$ T cells were sorted from *ERT2*$^{Cre}$*Rosa26*$^{YFP}$ and *ERT2*$^{Cre}$*Lkb1*$^{f/f}$*Rosa26*$^{YFP}$ mice, and cultured with 4-hydroxytamoxifen for 48 h. $CD4^+YFP^+$ T cells were sorted and analyzed for apoptosis and proliferation after the stimulation with anti-CD3+anti-CD28 for 48 h.(**h**) Intracellular phosphorylation of NF-κB p65 in $T_{reg}$ cells from *Foxp3*$^{Cre}$ and *Foxp3*$^{Cre}$*Tgfbr2*$^{f/f}$ mice, stimulated with or without IL-2 (n=3). (**i**) Intracellular phosphorylation of STAT4 in $T_{reg}$ cells from *Foxp3*$^{Cre}$ and *Foxp3*$^{Cre}$*Tgfbr2*$^{f/f}$ mice, stimulated with or without IL-12, respectively (n=3). (**j**) Intracellular phosphorylation of Smad2/3 in *Foxp3*$^{Cre}$ and *Foxp3*$^{Cre}$*Lkb1*$^{f/f}$ $T_{reg}$ cells supplemented with or without 4-ASA (n=3). Two-way ANOVA was used for statistical analyses in **c**, **d**, **e**, **h**, **i** and **j** (*$P$<0.05, **$P$<0.01); error bars represent s.d.; data represent at least two independent experiments.

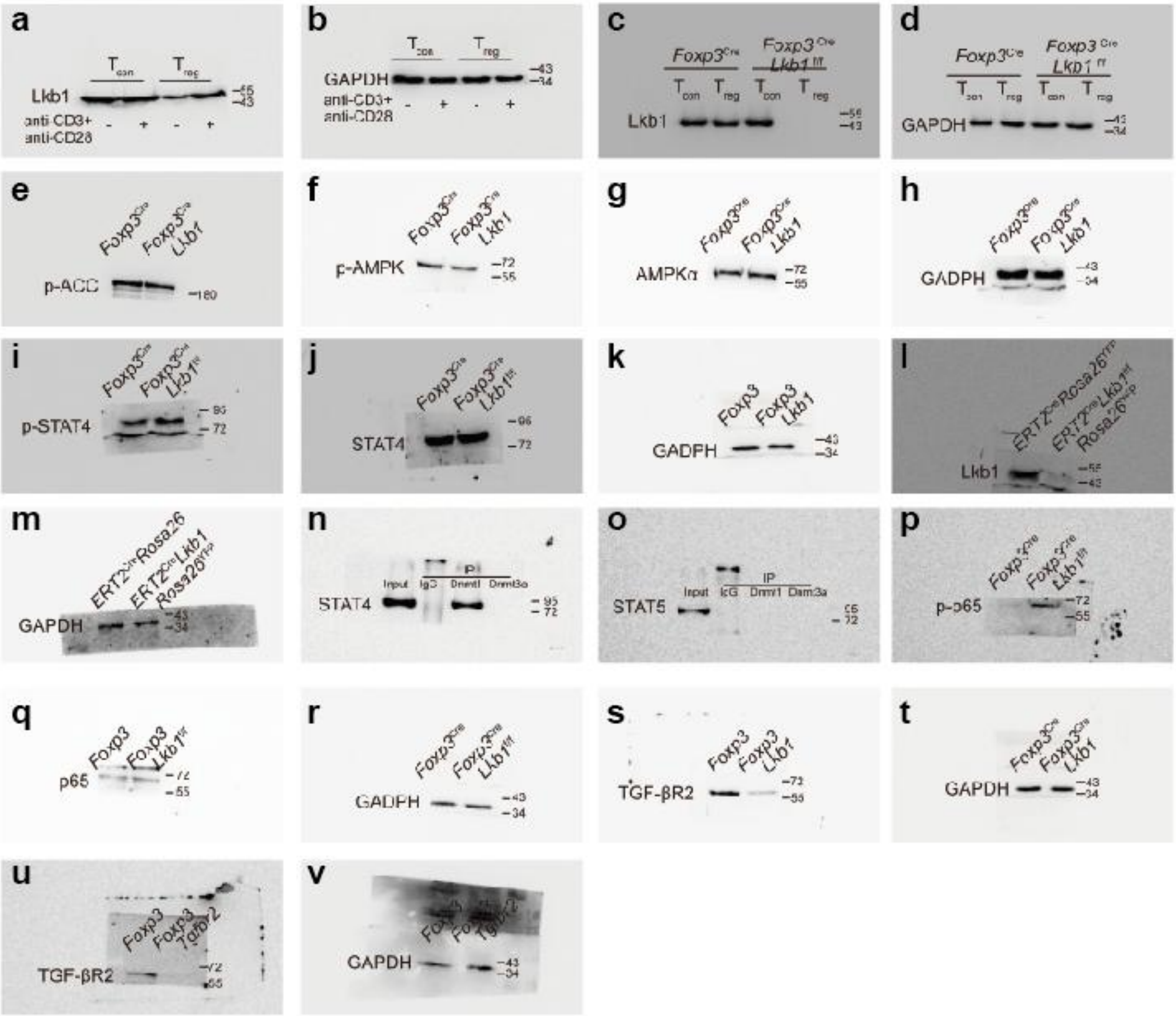


**Supplementary Figure 8. Uncropped scans of the western blots.** (**a,b**)-> Figure 1a.Lkb1 and GAPDH proteins in CD4$^{+}$YFP$^{-}$T$_{con}$ cells and CD4$^{+}$YFP$^{+}$T$_{reg}$ cells un-treated or stimulated in plates coated with anti-CD3 and anti-CD28 in the presence of IL-2 for 24 h.(**c,d**) ->Supplementary figure1b.Lkb1 protein was depleted in CD4$^{+}$YFP$^{+}$ T$_{reg}$ cells from 2-week-old *Foxp3*$^{Cre}$*Lkb1*$^{f/f}$ mice.(**e-h**) -> Supplementary figure 3a.Representative images of phosphorylated acetyl-coa carboxylase (ACC, Ser79), phosphorylated AMPKα (Thr172), total AMPKα, and GAPDH expression in *Foxp3*$^{Cre}$ and *Foxp3*$^{Cre}$*Lkb1*$^{f/f}$ T$_{reg}$ cells.(**i-k**) -> Supplementary figure 4b.Representative images of phosphorylated STAT4 (Tyr693), total STAT4 and GAPDH expression in *Foxp3*$^{Cre}$ and *Foxp3*$^{Cre}$*Lkb1*$^{f/f}$ T$_{reg}$ cells. (**l-m**) -> Supplementary figure 4e.Lkb1 protein was depleted in *ERT2*$^{Cre}$*Rosa26*$^{YFP}$ and *ERT2*$^{Cre}$*Lkb1*$^{f/f}$*Rosa26*$^{YFP}$T$_{reg}$ cells co-cultured with DCs supplemented with indicated cytokines and 4-hydroxytamoxifen.(**n,o**) ->Figure5c.STAT4 and STAT5 coprecipitation with Dnmt1 and Dnmt3awas analyzed by using nuclear extract from in vitro expanded T$_{reg}$ cells.(**p-r**) -> Supplementary figure 5c.Representative images of phosphorylated p65 (Ser536), total p65 and GAPDH expression in *Foxp3*$^{Cre}$ and *Foxp3*$^{Cre}$*Lkb1*$^{f/f}$ T$_{reg}$ cells.(**s,t**) ->Sup fig 7a. TGF-βR2 protein was decreased in Lkb1-deficient T$_{reg}$ cells compared with wild-type T$_{reg}$ cells. (**u,v**) -> Supplementary figure7b. TGF-βR2 protein was depleted in CD4$^{+}$YFP$^{+}$ T$_{reg}$ cells from *Foxp3*$^{Cre}$*tgfbr2*$^{f/f}$ mice.

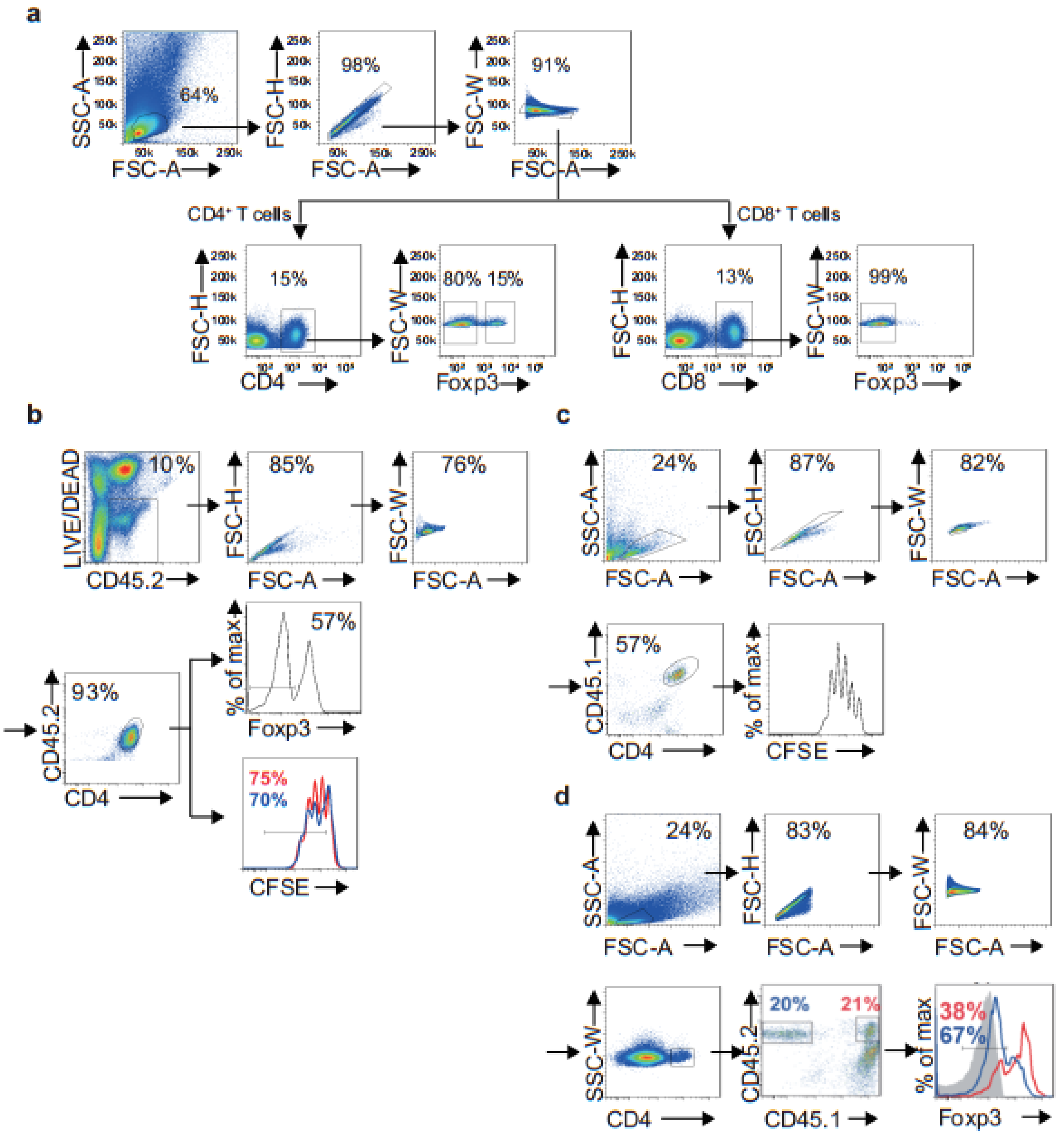


**Supplementary Figure 9. Flow cytometry gating strategies.** (**a**)-> Figure 2a-c,3a,4a,6a,6c,6f,g,7a-c,7f,,8b,8d,e,8h, Supplementary Figure 2b,2d,e,3c,d,4a,5b,5d,7c,7h-j. (**b**)-> Figure 4b-e,5e,6e, Supplementary Figure 4d,7g. (**c**)-> Figure 7d,8f,8j. (**d**)-> Figure 3c,8k.

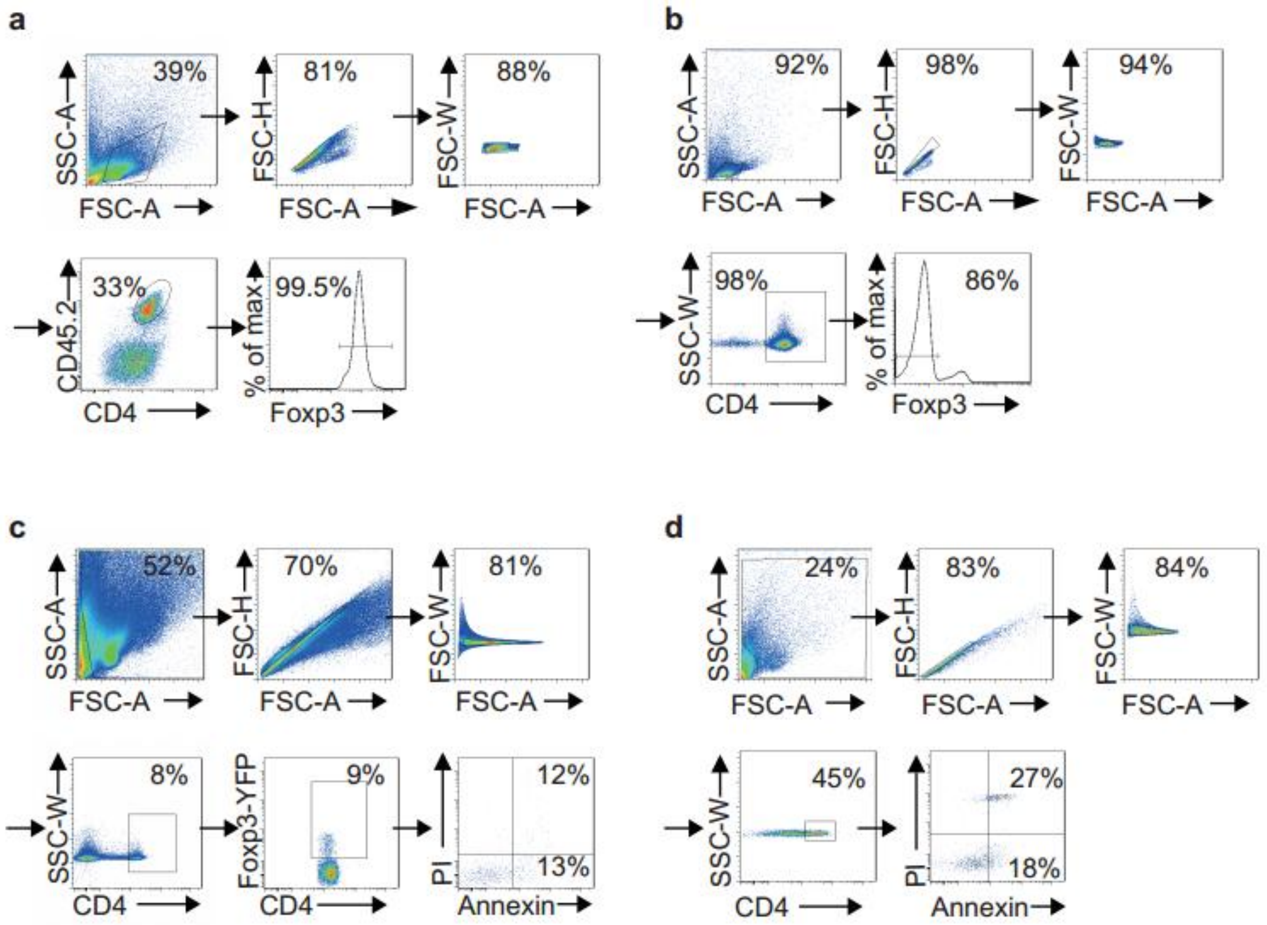


**Supplementary Figure 10. Flow cytometry gating strategies.** (**a**)-> Supplementary Figure 2d. (**b**)-> Figure 3b, Supplementary Figure 2c. (**c**)-> Supplementary Figure 2a,7f. (**d**)-> Supplementary Figure 4c.

| Tissue/cells | Target Population | Gating Strategy | Figure number |
|---|---|---|---|
| Spleen, lymph nodes and isolated $CD4^+$ cells ( Dynabeads Untouched Mouse CD4 Cells Kits) | $CD4^+Foxp3^-$ T cells | Immune cells/Singlets/$CD4^+$ / $Foxp3^-$ | Supplementary Figure 9a-> Figure 2a,b,c,7a,7b,8d Supplementary Figure 3d |
| | $CD8^+Foxp3^-$ T cells | Immune cells/Singlets/$CD8^+$ / $Foxp3^-$ | Supplementary Figure 9a-> Figure 2a,b,c, 7a,7b Supplementary Figure 3d |
| | $CD4^+Foxp3^+$ $T_{reg}$ cells | Immune cells/Singlets/$CD4^+$ / $Foxp3^+$ | Supplementary Figure 9a-> Figure 3a,4a,6a,6c,6f,6g,7c,7f,8b,8e,8h Supplementary Figure 2b,2d,e,3c,4a,5b,5d,7c,7h-j |
| Co-cultured cells | $CD4^+$ $CD45.2^+Foxp3^+$ $T_{reg}$ cells | $Live^+CD45.2^+$ cells/Singles/$CD4^+$/ CFSE/$Foxp3^+$ | Supplementary Figure 9b-> Figure 4b-e,5e,6e, Supplementary Figure 4d |
| Isolated $CD4^+Rosa^{YFP+}$ cells | $CD4^+$ T cells | Immune cells/Singlets/$CD4^+$ /CFSE | Supplementary Figure 9b-> Supplementary Figure 7g |
| Co-cultured cells | $CD4^+$ $CD45.1^+$ Tn cells | Immune cells/Singlets/$CD4^+$ $CD45.1^+$/ CFSE | Supplementary Figure 9c-> Figure 7d,8f,8j |
| Spleen and lymph nodes | $CD4^+CD45.2^+$/$CD45.1^+CD45.2^+Foxp3^+$ $T_{reg}$ cells | Immune cells/Singlets/$CD4^+$ / $CD45.1^+CD45.2^+$ and $CD45.2^+$/$Foxp3^+$ | Supplementary Figure 9d-> Figure 3c,8k |
| Isolated $CD4^+$ cells | $CD4^+$ $CD45.2^+Foxp3^+$ $T_{reg}$ cells | Immune cells/Singlets/$CD4^+$ $CD45.2^+$/ $Foxp3^+$ | Supplementary Figure 10a-> Supplementary Figure 2d |
| Isolated $CD4^+$ RosaYFP $^+$ cells | $CD4^+Foxp3^+$ $T_{reg}$ cells | Immune cells/Singlets/$CD4^+$ / $Foxp3^+$ | Supplementary Figure 10b-> Figure 3b, Supplementary Figure 2c |
| Spleen, lymph nodes | $CD4^+YFP^+$ $T_{reg}$ cells | Immune cells/Singlets/$CD4^+$ /$YFP^+$/ Annexin V-PI | Supplementary Figure 10c-> Supplementary Figure 2a |
| Isolated $CD4^+Rosa^{YFP+}$ cells | $CD4^+$ T cells | Immune cells/Singlets/$CD4^+$ /Annexin V-PI | Supplementary Figure 10c-> Supplementary Figure 7f |
| Co-cultured cells | $CD4^+$ $CD45.2^+$ $T_{reg}$ cells | Immune cells/Singlets/$CD4^+$ $CD45.2^+$/ Annexin V-PI | Supplementary Figure 10d-> Supplementary Figure 4c |

**Supplementary Table 1. Flow cytometry gating strategies.**
